# Greenness and averted mortality in 390 cities in China (2000–2020)

**DOI:** 10.1016/j.lanwpc.2024.101283

**Published:** 2025-01-16

**Authors:** John S. Ji, Zheng Tao, Hui Miao, Tom Cole-Hunter, Xuecao Li, David Rojas-Rueda, Wenjia Cai

**Affiliations:** a Vanke School of Public Health, Tsinghua University, Beijing, China; b Section of Environmental Health, Department of Public Health, University of Copenhagen, Copenhagen, Denmark; c College of Land Science and Technology, China Agricultural University, Beijing, China; d Department of Environmental and Radiological Health Sciences, Colorado State University, Fort Collins, CO, USA; e Colorado School of Public Health, Colorado State University, 1601 Campus Delivery, Fort Collins, CO, 80523, USA; f Department of Earth System Science, Tsinghua University, Beijing, China

**Keywords:** Green space, Health impact, China, Urban health, Healthy cities

## Abstract

**Background:**

China's growth over recent decades rapidly transformed the urban landscapes. Green spaces provide numerous health benefits including acting as nature-based solutions for climate change risks. Our study aims to track greenness trends in urban areas in China and quantify the health impact of greenness on adult mortality.

**Methods:**

In China, we mapped the urban human population distribution aged 20 and above with a 1 km grid (30 arc-second) and used satellite-based remote sensing to measure green space over time to create population-weighted normalized difference vegetation index (NDVI). We tracked changes in greenness in the urban area over time and created a spatial–temporal map. Based on counterfactual scenarios, we calculated averted deaths attributed to NDVI changes from 2000 to 2020.

**Findings:**

We analyzed and mapped 390 cities or urban areas in China, covering an urban population of nearly 500 million. We found population-weighted NDVI exhibiting an overall increase from 2000 to 2020 for most cities. Our analysis calculated urban areas that experienced decrease in urban NDVI from 2000 to 2010 could have had an estimated 9951 additional deaths annually (95% CI: 3346–18,106), while increase in NDVI from 2010 to 2020 could have averted an estimated 37,653 deaths annually (95% CI: 26,327–60,135). If the NDVI were increased to the target level in 2000 and 2010, the number of deaths would be reduced by 110,976 (95% CI: 82,010–171,561) and 118,330 (95% CI: 87,362–183,283), respectively.

**Interpretation:**

Greenness has increased in most urban in China since 2000. Considering the ongoing impacts of climate change and urbanization, sustained efforts in greenness management could serve as an effective resilience factor for protecting population health.

**Funding:**

Natural Science Foundation of Beijing (IS23105), 10.13039/501100001809National Natural Science Foundation of China (82250610230, 72061137004), 10.13039/100004423World Health Organization (2024/1463606-0), Research Fund Vanke School of Public Health10.13039/501100004147Tsinghua University (2024JC002).

**Research in context**

**Evidence before this study**

We conducted literature searches in PubMed, Scopus, and Google Scholar databases until October 8, 2024, to estimate the impact of green exposure on health without language or publication date restrictions. Our terms included “green space” OR “greenness” OR “green exposure” OR “NDVI” AND “mortality” OR “premature mortality” AND “health impact assessment” OR “health impact.” We excluded studies that only had abstracts, systematic reviews, and studies that did not use NDVI as the primary indicator. A total of 16 studies were retrieved. Twelve were cohort studies, four were ecological studies, and six were conducted in China. Previous studies estimated the health effects of green exposure for several selected cities, such as Philadelphia, Barcelona, Vienna, Madrid, London, Stockholm, and Hong Kong. Two large city-level studies estimated the health effects of green space exposure. One study assessed how many adult residents' deaths from natural causes could be prevented in 978 European cities if the green space area was increased to the recommendation of the World Health Organization; another study used difference-in-differences analysis to assess the association between green space and mortality in China in the years 2000, 2010, and 2019 and explored the potential mediating roles of air pollution and temperature.

**Added value of this study**

Based on high-resolution NDVI data, this study quantitatively assessed the changes in greenness and their impact on public health across 390 cities in China. Our results indicate that the greenness levels in most cities have shown a significant upward trend. Notably, the contribution of greenness growth in the northern regions to reducing mortality is particularly prominent, surpassing that of the southern regions. Besides, a considerable proportion of deaths in Chinese cities could be avoided annually by increasing greenness. To our knowledge, this is the first health impact assessment study to estimate the potential impact of long-term greenness changes on averted mortality in China.

**Implications of all the available evidence**

Our study tracked greenness changes over time and presented estimations of averted mortality along with simulated preventable deaths in a counterfactual scenario where greenness increased. China being one of the most populous places, these findings have implications for urban policy-making, as we provide a comprehensive assessment of the impact of greenness on health and tracking of this indicator for The Lancet Countdown Report against the backdrop of climate change and rapid urbanization, our research underscores the importance of green space management as an effective public health protection strategy.

## Introduction

Urban green space is associated with positive benefits on mental health, non-communicable diseases, and mortality.^1^ Having accessible green space in a community can foster social interaction and create a positive environment for physical activity,^2^ and the health benefits are amplified in lower social economic or deprived communities.^3^ Green space may be an effect modifier for climate hazards. In the Lancet Countdown, the urban green space is an indicator for climate change adaptation, it has interactions with non-optimal temperature and air pollution-related mortalities.^2^^,^^4^^,^^5^ In the past few decades, China has seen an accelerated trend of rural residents moving to cities, with estimated 65% of the Chinese population now living in cities.^6^ The growth of cities has led to the expansion of urban areas and increased population density, resulting in a surge of built-environment land use and impervious surfaces. This change in landscape has led to increased impervious surfaces, which prevent water from infiltrating the soil and trap heat during the day, creating hot nights. Green spaces are vital for adapting to climate change and mitigating the urban heat island effect. They help reduce city temperatures and combat the negative effects of air pollution, heat, and noise. Additionally, green spaces promote physical activity, mental well-being, and social interaction, benefiting all community residents.

Over the past two decades, China's rapid urbanization process has been accompanied by significant changes in greenness. Across the board, the average green space coverage in cities studied has steadily increased, with the highest per capita gross domestic product (GDP) areas demonstrating the strongest independent contribution to green space coverage.^7^ Despite the well-documented health benefits of green spaces in natural settings, the impact of changes in urban green spaces on averted mortalities in China is dynamic and has not been systematically assessed.

The primary objective of our study is to evaluate the changes in urban green spaces across 390 cities in China and to investigate the potential impact of such changes on mortality. Furthermore, we aimed to establish a relationship between greenness and averted deaths in urban areas. Our research aims to generate meaningful policy implications and interventions that can increase accessibility to green spaces and promote health in urban environments.

## Methods

### Definition of urban areas and cities

Our study included all urban areas in China, encompassing 390 cities (Appendix p3, Table S1). China has a four-tier city system based on specific administrative levels: provincial-level municipalities directly under the central government, prefecture-level cities, county-level cities, and towns. Since county-level cities are part of prefecture-level cities and are geographically located within them, to avoid double counting, we selected all prefecture-level administrative units (including prefecture-level cities, regions, autonomous prefectures, and leagues) and 6 provincial-level units (including municipalities and special administrative regions) as the study areas. The administrative boundaries of these units were obtained from the China Ministry of Civil Affairs.^8^

The urban areas within each city were delineated using extent polygons from the Global Rural-Urban Mapping Project (GRUMP) (Appendix p3, Figure S1).^9^^,^^10^ GRUMP delineates urban areas starting from global nighttime light data, which are transformed into polygons representing urban extents, encompassing all urban areas with populations of 1000 or more.

We divided the cities, municipalities, and special administrative regions into seven geographic regions: North China (including Beijing, Tianjin, Hebei, Shanxi, and Inner Mongolia), Northeast China (including Liaoning, Jilin, and Heilongjiang), East China (including Shanghai, Jiangsu, Zhejiang, Anhui, Fujian, Jiangxi, Shandong, and Taiwan), Central China (including Henan, Hubei, and Hunan), South China (including Guangdong, Guangxi, Hainan, Hong Kong, and Macao), Southwest China (including Chongqing, Sichuan, Guizhou, Yunnan, and Tibet), and Northwest China (including Shaanxi, Gansu, Qinghai, Ningxia, and Xinjiang).

### Population and mortality

We collected population data from the Gridded Population of World Version 4 (GPWv4), Revision 11.^11^ The data source utilized in this study provided information on the distribution of the global human population across 30 arc-second grid cells (roughly equivalent to 1 km) for the years 2000, 2005, 2010, 2015, and 2020. To calculate the total population within the urban areas of each city, we summed the populations of the relevant grid cells. We also used national census statistics from 2000, 2010, and 2020 to determine the population aged 20 and above, based on age-specific population proportions (Appendix p3, Table S1). Since province-level mortality rates data was for the whole population, we used national-level mortality rates in 2000 (5.5‰), and 2010 (4.3‰) for population aged 20 and above, based on national annual statistics. Our study seeks to provide a comprehensive description of the relationship between population density, age demographics, and mortality rates in urban areas.^12^, ^13^, ^14^

### Exposure to greenness

To assess the levels of green space in urban areas, we employed satellite remote sensing to measure the NDVI, a widely used metric for monitoring vegetation. It is calculated as the ratio of the difference in the surface reflectance intensities of red and infrared radiation to the sum of their intensities. The resulting NDVI values range from −1 to 1, with higher positive NDVI values generally indicating a higher density of green vegetation and a higher level of greenness.

We utilized NDVI images from the Moderate Resolution Imaging Spectroradiometer (MODIS) sensor to estimate green exposure. MOD13Q1 data are provided every 16 days at 250-m spatial resolution.^15^ We obtained all MOD13Q1 images from 2000, 2005, 2010, 2015, to 2020 and calculated the median NDVI value on a pixel basis to obtain an NDVI median layer for each year. The MODIS products were pre-processed with atmospheric correction and cloud-free coverage, utilizing the historical time series climatology to replace cloud pixels, thus enhancing data reliability. Additionally, considering the possibility that blue spaces have independent beneficial effects on health, water bodies were masked out from the analysis using the MOD44W.005 data product.^16^ To ensure the accuracy and reliability of the NDVI data, we conducted a preprocessing step to exclude potential outliers. This involved utilizing the quality assurance (QA) flags provided in the MOD13Q1 dataset to identify and mask pixels affected by clouds, aerosols, or other atmospheric disturbances. Additionally, we applied statistical filters to remove anomalous NDVI values that fell outside the expected range for vegetated surfaces (e.g., NDVI < −0.2 or NDVI >1.0). Temporal consistency checks were also performed to detect and exclude sudden, uncharacteristic changes in NDVI values for individual pixels.

We calculated population-weighted NDVI for each city and province. This involved weighting the NDVI values for each pixel by the corresponding population density and then averaging across all pixels to obtain an overall population-weighted NDVI value for the city or province. The equation used to calculate the population-weighted NDVI value for each city and province was:

$$population-weighted\;NDVI=\frac{\sum_{i=1}^{n}\left(NDVI_{i}\;\times \;Pop_{i}\right)}{\sum_{i=1}^{n}Pop_{i}}$$

where n is the number of pixels in an urban area with valid NDVI data in a city or a province. To assess the trend of urban greenness over the past decade, we utilized population-weighted NDVI and performed linear regression analysis (appendix p4) for each city and province from 2000 to 2020. A significant increase or decrease in the trend of greenness was determined if the coefficient of the slope was positive or negative with a p-value less than 0.05. Conversely, if the p-value was greater than 0.05, the trend was considered as showing no significant change.

### Health impact assessment

We conducted a quantitative health impact assessment on 390 cities to estimate the effect of greenness averted mortality among the population aged 20 years and above in China. Initially, we estimated the averted deaths to be attributable to changes in greenness over a 20-year period in China. Additionally, based on previous health assessment frameworks,^17^^,^^18^ we evaluated preventable deaths due to the difference between actual exposure and counterfactual scenarios. The procedures are as follows: 1) The level of green exposure was estimated using the NDVI. 2) We obtained the exposure-response function (ERF) from existing literature to quantify the relationship between green exposure and mortality (Appendix p5, Table S2). 3) We calculated the changes in greenness between 2010 and 2000, and 2020 and 2010. Then, we estimated the baseline green exposure and calculated the differences between the actual exposure and counterfactual scenarios in 2000 and 2010. 4) We estimated the relative risk (RR) corresponding to these exposure changes or differences. Based on the exposure differences and RR, we calculated the attributable fraction (AF), which was used to estimate the mortality burden. 5) We conducted uncertainty analysis using 10,000 Monte Carlo simulations to obtain point estimates and confidence intervals (CIs). 6) We calculated the deaths for each city and aggregated the results across all cities. We used Google Earth Engine, ArcGIS (version 10.1), and R (version 4.3.2) for the analysis.

In this study, averted mortality refers to the number of deaths among adults aged 20 and above that could potentially be prevented by increasing exposure to urban green space to meet specific target levels of NDVI through policy actions or interventions. Preventable mortality is used interchangeably with ‘averted mortality’ in our context and denotes deaths that could theoretically be avoided in a hypothetical scenario where NDVI reaches an ideal target level.

We estimated the deaths attributable to NDVI changes (or difference). Mortality, M is expressed as:

$$M=y_{0}\;\times \;Pop\;\times \;AF$$

where $y_{0}$ is the national-level annual all-cause mortality rate for population aged 20 and above in 2000 and 2010, Pop is the population size and AF is the attributable fraction of NDVI changes (or difference). AF is calculated via the relative risk (RR), which represents the decrease of risk of mortality resulting from the NDVI changes (or difference). AF is calculated as:

$$AF=\frac{RR\;-\;1}{RR}$$

A 0.1-unit increase of NDVI could reduce mortality risk by 4%^19^ i.e. $RR_{0.1-unit-increase}=0.96\;\left(95\%\;CI\;0.94-0.97\right)$. The RR of NDVI change is calculated as:

$$RR_{change}={RR_{0.1-unit-increase}}^{change/0.1}$$

where change is NDVI in 2010 subtracted by that in 2000, or NDVI in 2020 subtracted by that in 2010. Similarly, we calculated the RR for the ‘difference’ between the counterfactual exposure and the actual exposure level:

$$RR_{difference}=\exp\;\left(\left(\frac{\ln\left(RR_{0.1-unit-increase}\right)}{0.1}\right)*\left(\;difference\right)\right)$$

where difference is the difference between the actual NDVI and the counterfactual target.

### Counterfactual scenarios of green exposure

Counterfactual scenarios of green exposure are established to estimate the preventable deaths in health impact assessments. We set the target level of greenness for the counterfactual exposure as the 75th percentile of NDVI at the city level. This standard is chosen under the assumption that 75% of cities are exposed to NDVI levels lower than the counterfactual level.

### Uncertainty analysis

Uncertainty analyses were conducted on 7 regions in China to assess the impact of uncertainty distribution estimates of NDVI change (or difference), population, and ERF (Appendix p6, Figure S2) on the mortality counts. Subsequently, we constructed an uncertainty distribution and obtained point estimates and 95% CI using 10,000 Monte Carlo simulations for the final estimates (Appendix p6, Table S3).

### Sensitivity analysis

We performed sensitivity analyses to assess the effect of changes in model variables on the final mortality estimates. We tested the effects of using different ERFs, as well as the effects of unweighted NDVI and the mean NDVI from June to August (i.e. the greenest period of the year) on mortality estimates (Appendix p7, Tables S4 & S5).

### Ethics approval

All data were publicly available and de-identified, hence no ethical approval was required.

### Role of the funding source

The funding agency of the study had no role in the study design, data collection, data analysis, data interpretation, or writing of the manuscript.

## Results

According to our analysis of the NDVI which measures greenness levels in urban areas, there has been an overall increase in greenness in China over the past 20 years. However, there was a decrease in greenness from 2000 to 2010, followed by an increase from around 2010 to 2020. Out of 390 cities in China, 48 cities experienced a significant increase in population-weighted NDVI in the urban areas from 2000 to 2020, while six cities showed a significant decrease. These cities include Shantou in Guangdong province, Putian and Quanzhou in Fujian province, Chengdu and Meishan in Sichuan province, and Dandong in Liaoning. On a provincial level, Beijing, Chongqing, Hong Kong, Guizhou province, Shaanxi province, and Xinjiang province saw an increase in population-weighted NDVI in urban areas between 2000 and 2020, while other provinces did not show a significant change in urban greenness level (Table 1).

**Table 1:** NDVI in provinces, provincial capitals and major cities in China, 2000–2020.

Area	Unweighted NDVI							Population-weighted NDVI					
	2000	2020	Diff^a^	Trend^b^	City ranking within China	City ranking within area	Annual preventable death per 100,000 inhabitants	2000	2020	Diff^a^	Trend^b^	Ranking within China	Ranking within area
**North China**													
**Beijing**	0.292	0.378	0.109	No change	283	8	−27.9	0.236	0.336	0.114	Increase	291	10
**Tianjin**	0.242	0.292	0.057	No change	338	24	−15.1	0.212	0.268	0.070	No change	342	20
**Hebei**	0.296	0.355	0.064	No change			−10.4	0.277	0.327	0.056	No change		
Shijiazhuang	0.352	0.369	0.013	No change	288	10	−7.4	0.288	0.323	0.033	No change	300	12
Handan	0.279	0.383	0.083	No change	278	7	−16.7	0.276	0.354	0.069	No change	265	6
**Shanxi**	0.246	0.342	0.091	No change			−14.6	0.218	0.285	0.071	No change		
Taiyuan	0.207	0.308	0.086	Increase	328	19	−15.1	0.153	0.220	0.061	Increase	369	27
Yuncheng	0.325	0.438	0.127	No change	236	4	−19.0	0.313	0.416	0.126	No change	186	2
**Inner Mongolia**	0.271	0.290	0.039	No change			−8.3	0.202	0.232	0.042	No change		
Hohhot	0.211	0.244	0.036	No change	358	27	−4.7	0.184	0.203	0.022	No change	377	33
Chifeng	0.240	0.296	0.076	No change	335	21	−7.5	0.212	0.253	0.059	No change	352	23
**Northeast China**													
**Liaoning**	0.330	0.323	0.042	No change			−3.0	0.281	0.294	0.040	No change		
Shenyang	0.306	0.287	0.034	No change	339	31	−8.1	0.231	0.266	0.044	No change	345	34
Dalian	0.335	0.347	0.044	No change	303	20	−8.0	0.275	0.306	0.046	No change	316	24
**Jilin (province)**	0.386	0.395	0.071	No change			−8.6	0.285	0.321	0.069	No change		
Changchun	0.291	0.338	0.071	No change	310	22	−15.0	0.231	0.297	0.077	No change	324	28
Jilin (city)	0.438	0.456	0.074	No change	220	5	−11.4	0.265	0.315	0.074	Increase	305	18
**Heilongjiang**	0.412	0.404	0.018	No change			−4.9	0.318	0.331	0.027	No change		
Harbin	0.383	0.389	0.016	No change	274	15	−4.3	0.285	0.301	0.023	No change	320	26
Qiqihar	0.355	0.339	0.009	No change	309	21	−4.6	0.290	0.307	0.028	No change	314	23
**East China**													
**Shanghai**	0.420	0.365	0.024	No change	292	96	−6.9	0.331	0.325	0.054	No change	299	95
**Jiangsu**	0.437	0.454	0.033	No change			−1.8	0.378	0.378	0.035	No change		
Nanjing	0.419	0.430	0.064	No change	243	76	−11.4	0.342	0.372	0.100	No change	241	75
Suzhou	0.424	0.366	0.028	No change	290	94	5.0	0.380	0.338	0.031	No change	289	94
**Zhejiang**	0.485	0.472	0.001	No change			1.3	0.439	0.407	−0.012	No change		
Hangzhou	0.508	0.479	0.008	No change	194	56	3.2	0.471	0.386	−0.014	No change	227	66
Wenzhou	0.486	0.487	−0.006	No change	186	52	−0.8	0.424	0.417	−0.019	No change	185	48
**Anhui**	0.454	0.490	0.031	No change			−4.6	0.395	0.430	0.052	No change		
Hefei	0.418	0.445	0.059	No change	232	69	0.9	0.349	0.386	0.114	No change	228	67
Fuyang	0.477	0.532	0.031	No change	124	26	−6.6	0.449	0.500	0.040	No change	93	21
**Fujian**	0.556	0.552	0.009	No change			4.7	0.450	0.417	−0.023	No change		
Fuzhou	0.534	0.549	0.033	No change	113	22	−2.6	0.418	0.444	0.012	No change	151	37
Quanzhou	0.478	0.442	−0.019	Decrease	233	70	13.6	0.406	0.341	−0.036	Decrease	287	93
**Jiangxi**	0.509	0.509	0.034	No change			4.5	0.409	0.385	0.006	No change		
Nanchang	0.431	0.400	0.029	No change	261	83	4.2	0.327	0.308	0.018	No change	313	97
Ganzhou	0.543	0.551	0.044	No change	107	21	5.6	0.430	0.410	0.021	No change	199	52
**Shandong**	0.350	0.401	0.084	No change			−8.0	0.317	0.360	0.079	No change		
Jinan	0.317	0.398	0.121	No change	267	88	−15.8	0.262	0.346	0.132	No change	278	90
Qingdao	0.312	0.332	0.041	No change	313	97	−8.5	0.261	0.305	0.053	Increase	317	98
**Taiwan**	0.565	0.585	0.043	No change			−2.7	0.477	0.496	0.046	No change		
Taipei	0.568	0.602	0.051	No change	57	13	−8.1	0.485	0.523	0.052	No change	71	15
New Taipei	0.693	0.733	0.044	Increase	5	2	−6.4	0.457	0.487	0.041	No change	110	28
**Central China**													
**Henan**	0.391	0.477	0.111	No change			−8.8	0.356	0.402	0.084	No change		
Zhengzhou	0.324	0.372	0.055	No change	286	49	−7.4	0.287	0.312	0.061	No change	310	49
Nanyang	0.402	0.502	0.135	No change	162	27	−9.8	0.387	0.450	0.115	No change	142	17
**Hubei**	0.460	0.517	0.065	No change			−1.0	0.366	0.392	0.045	No change		
Wuhan	0.362	0.379	0.045	No change	282	48	−11.8	0.282	0.336	0.075	No change	292	48
Huanggang	0.469	0.506	0.066	No change	155	24	2.7	0.435	0.435	0.020	No change	160	24
**Hunan**	0.491	0.548	0.073	No change			−7.2	0.408	0.434	0.052	No change		
Changsha	0.459	0.489	0.079	No change	181	30	−7.1	0.323	0.351	0.074	No change	270	46
Hengyang	0.461	0.534	0.087	No change	122	15	−5.6	0.414	0.443	0.069	No change	152	20
**South China**													
**Guangdong**	0.500	0.525	0.055	No change			−3.0	0.405	0.420	0.046	No change		
Guangzhou	0.451	0.469	0.070	No change	204	48	−9.1	0.314	0.353	0.085	No change	267	54
Shenzhen	0.436	0.478	0.065	No change	197	47	−14.6	0.366	0.411	0.073	No change	196	47
**Guangxi**	0.527	0.564	0.032	No change			−3.8	0.429	0.468	0.031	No change		
Nanning	0.503	0.526	0.005	No change	131	40	−2.2	0.343	0.388	0.044	No change	225	50
Yulin	0.486	0.520	0.023	No change	139	42	−4.5	0.450	0.488	0.015	No change	109	33
**Hainan**	0.584	0.604	0.055	No change			0.4	0.502	0.479	0.034	No change		
Haikou	0.521	0.506	0.028	No change	154	44	6.6	0.438	0.407	0.045	No change	203	49
Sanya	0.650	0.620	0.004	No change	38	15	10.6	0.587	0.530	−0.002	No change	67	26
**Hong Kong**	0.637	0.689	0.059	Increase	11	5	−15.6	0.435	0.495	0.049	Increase	99	32
**Macao**	0.400	0.400	0.081	No change	260	53	−1.4	0.232	0.237	0.095	No change	361	55
**Southwest China**													
**Chongqing**	0.526	0.570	0.019	No change	85	30	−10.2	0.439	0.480	0.029	Increase	114	35
**Sichuan**	0.525	0.582	0.024	Increase			−9.4	0.483	0.474	−0.014	No change		
Chengdu	0.561	0.498	−0.074	No change	167	42	5.6	0.486	0.416	−0.039	Decrease	187	44
Nanchong	0.456	0.551	0.026	No change	109	34	−12.7	0.427	0.460	−0.010	No change	128	37
**Guizhou**	0.497	0.585	0.083	Increase			−13.5	0.450	0.500	0.055	Increase		
Guiyang	0.468	0.518	0.053	Increase	142	37	−11.0	0.414	0.454	0.043	Increase	136	39
Bijie	0.472	0.570	0.103	No change	83	29	−16.2	0.444	0.525	0.093	No change	70	23
**Yunnan**	0.508	0.536	0.055	No change			−4.0	0.396	0.415	0.043	No change		
Kunming	0.445	0.460	0.044	No change	217	46	−7.1	0.296	0.330	0.059	No change	294	48
Qujing	0.453	0.508	0.067	No change	151	39	−2.8	0.424	0.446	0.037	No change	146	41
**Tibet**	0.220	0.222	0.012	No change			3.0	0.188	0.174	0.010	No change		
Lhasa	0.182	0.169	0.007	No change	380	52	3.6	0.181	0.163	0.011	No change	384	52
Shigatse	0.143	0.146	0.013	No change	384	53	1.1	0.156	0.151	0.010	No change	385	53
**Northwest China**													
**Shaanxi**	0.348	0.464	0.097	Increase			−17.0	0.335	0.413	0.053	Increase		
Xi'an	0.386	0.473	0.081	No change	201	12	−18.2	0.291	0.374	0.037	Increase	238	16
Weinan	0.357	0.456	0.107	No change	219	15	−15.0	0.344	0.427	0.089	No change	172	11
**Gansu**	0.222	0.306	0.065	Increase			−15.1	0.224	0.292	0.047	No change		
Lanzhou	0.205	0.241	0.035	No change	361	45	−8.9	0.191	0.232	0.030	No change	365	52
Tianshui	0.286	0.464	0.101	Increase	213	14	−38.5	0.267	0.454	0.110	Increase	137	8
**Qinghai**	0.200	0.215	0.019	No change			−6.0	0.226	0.247	0.028	No change		
Xining	0.291	0.284	0.025	No change	341	33	−7.0	0.226	0.254	0.039	No change	351	43
Haidong	0.258	0.279	0.012	No change	345	35	−1.0	0.259	0.271	0.000	No change	340	39
**Ningxia**	0.171	0.225	0.042	Increase			−14.7	0.192	0.250	0.059	No change		
Yinchuan	0.186	0.232	0.038	No change	364	46	−13.9	0.197	0.248	0.059	No change	356	47
Wuzhong	0.180	0.219	0.034	Increase	370	50	−13.0	0.220	0.273	0.056	No change	339	38
**Xinjiang**	0.233	0.264	0.020	Increase			−8.0	0.256	0.285	0.015	Increase		
Urumchi	0.247	0.271	0.028	No change	347	37	−14.1	0.223	0.281	0.038	No change	334	34
Kashgar	0.245	0.261	−0.008	No change	351	39	4.0	0.285	0.265	−0.033	No change	347	40

a Diff: NDVI in 2020 subtracted by NDVI in 2000.

b Trends of NDVI from 2000 to 2020 was calculated using linear regression. “No change” means there was no significant change in the two decades.

**Fig. 1:**
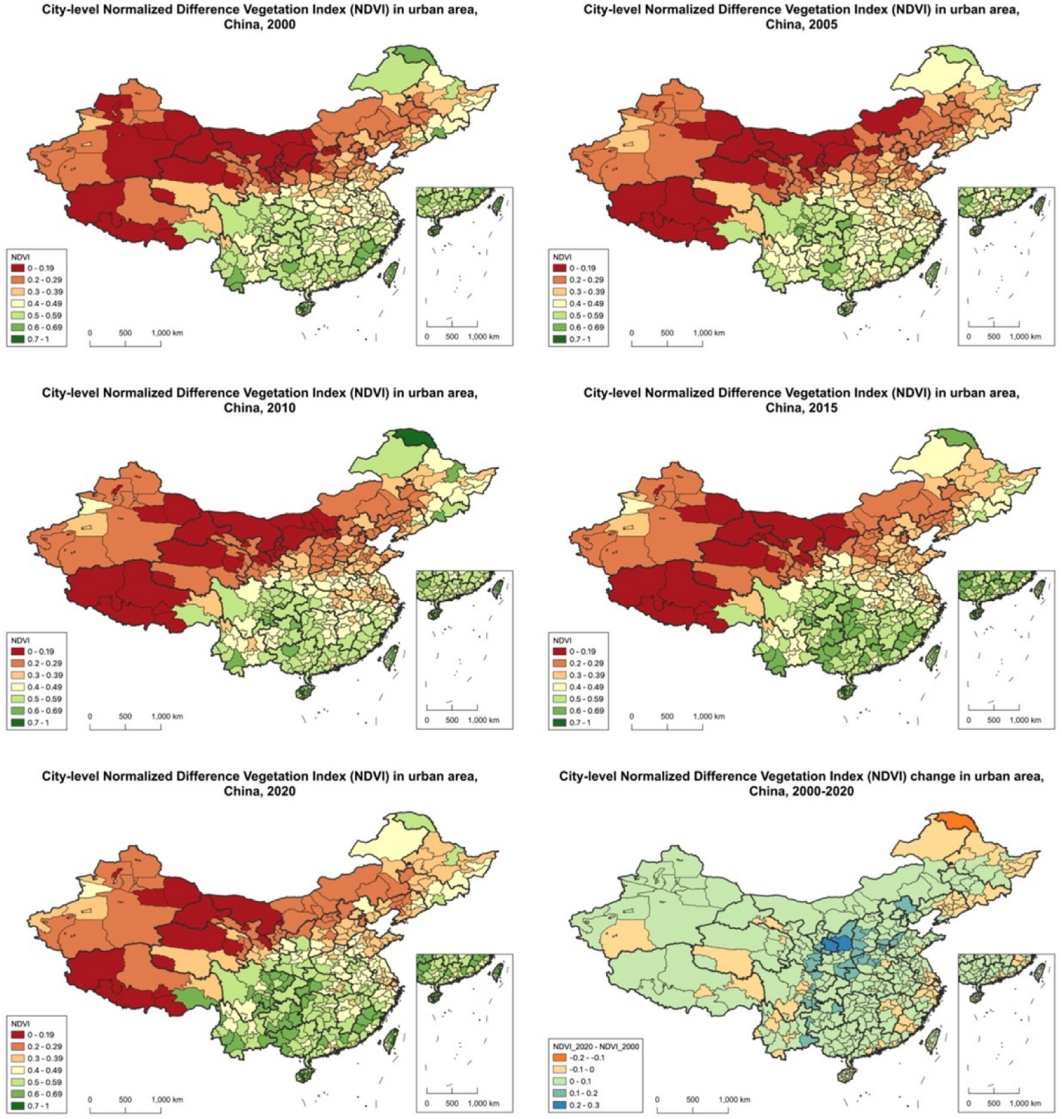
**Distribution of greenness exposure across cities in China, measured by unweighted NDVI, 2000–2020**.

**Fig. 2:**
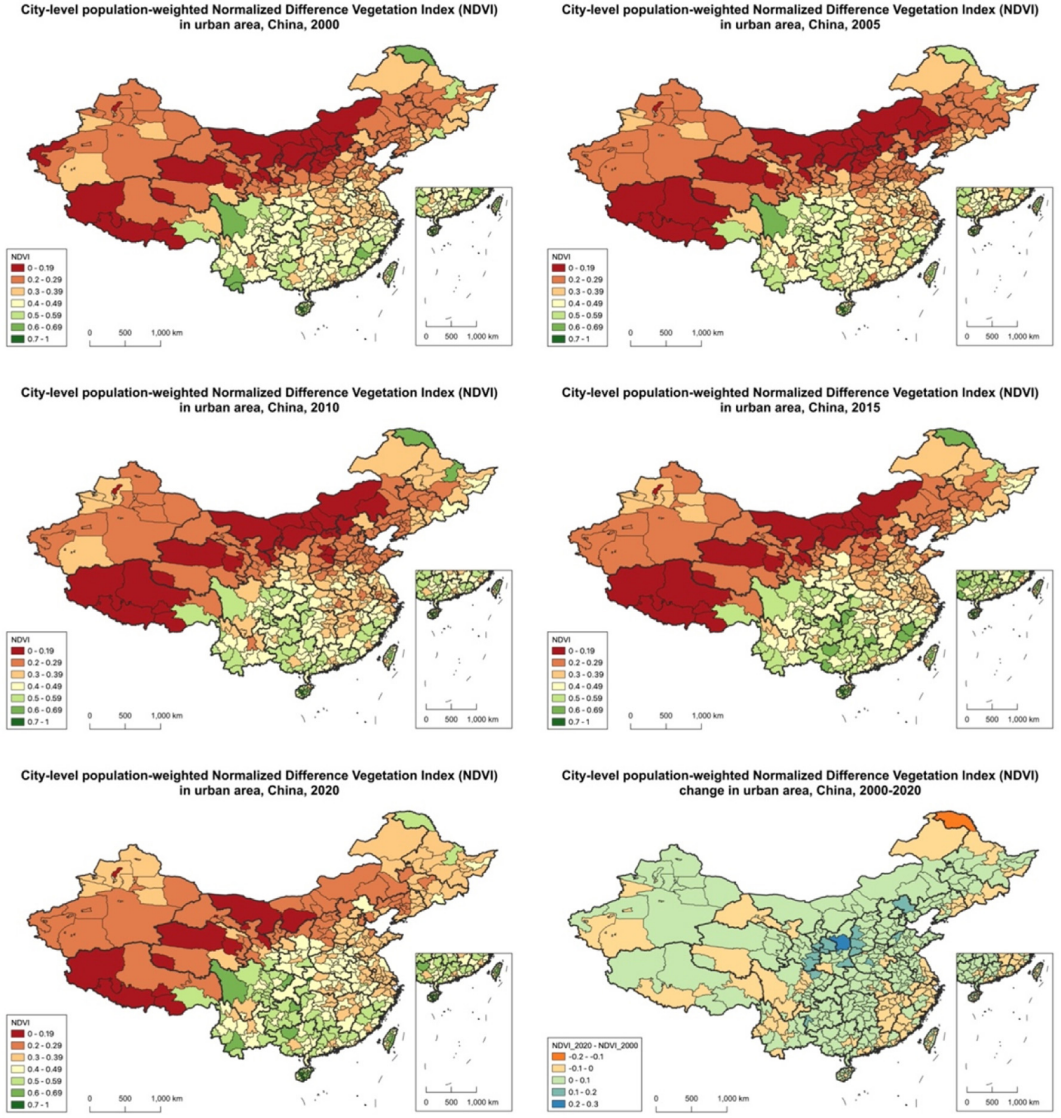
**Distribution of greenness exposure across cities in China, measured by population-weighted NDVI, 2000–2020**.

A The trends of urban green space measured by population-weighted NDVI and unweighted NDVI were generally consistent, but with some differences. When comparing greenness measured by weighted results, unweighted NDVI in 11 more cities (for a total of 59 cities) showed a significant increase in the past two decades. Additionally, unweighted NDVI in Shantou, Putian, Quanzhou, Ningbo (in Zhejiang province), and Xiamen (in Fujian province) demonstrated a decreasing trend (Figs. 1 and 2).

**Fig. 3:**
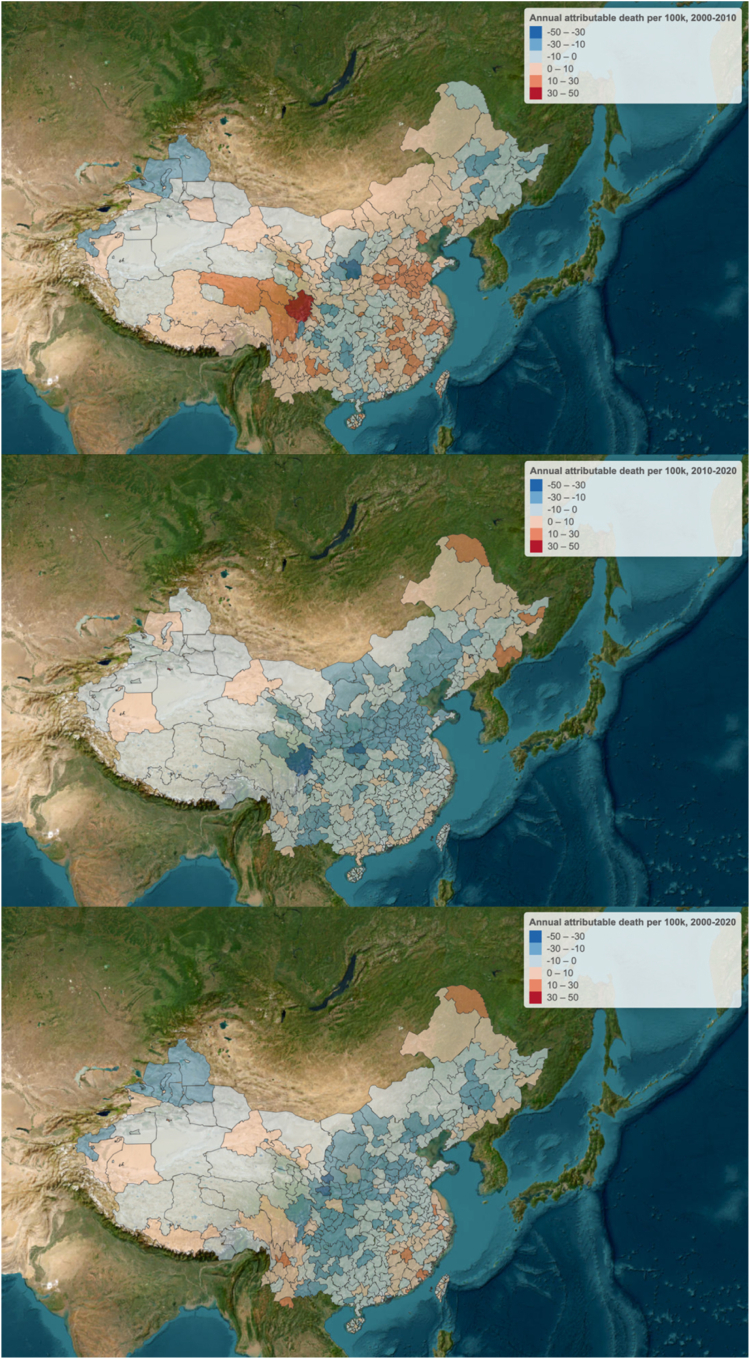
**Annual attributable death to NDVI per 100,000 population, 2000–2020**.

We estimated, across the 390 cities in China, that the decrease in urban NDVI from 2000 to 2010 could have caused 9951 additional deaths annually (95% CI: 3346–18,106), while the increase of NDVI from 2010 to 2020 could have averted an estimated 37,653 deaths annually (95% CI: 26,327–60,135) (Table 2). We also observed that the averted deaths tended to be higher in northern cities (Fig. 3). According to the counterfactual scenarios, 292 cities were exposed to below target greenness levels. Increasing the NDVI to the target levels in the year 2000 could have prevented 110,976 (95% CI 82,010–171,561) deaths, while in 2010, it could have prevented 118,330 (95% CI 87,362–183,283) deaths (Appendix p7, Table S4).

**Table 2:** Annual mortality attributable to NDVI change in provinces, provincial capitals and major cities in China.

Area	2000–2010				2010–2020			
	Population (2000)	Mortality	95% CI-lower	95% CI-upper	Population (2010)	Mortality	95% CI-lower	95% CI-upper
**Total**	**497,231,980**	**9951**	**3346**	**18,106**	**455,568,630**	**−37,653**	**−60,135**	**−26,327**
**North China**	63,775,078	1800	1102	2862	79,983,172	−11,073	−17,104	−8177
**Beijing**	10,710,832	−308	−498	−222	16,545,554	−2683	−4144	−1984
**Tianjin**	6,500,178	90	70	122	9,197,794	−1072	−1656	−793
**Hebei**	24,353,203	1487	1124	2198	28,270,942	−4019	−6203	−2972
Shijiazhuang	4,422,108	280	212	413	5,246,066	−607	−935	−450
Handan	3,535,171	250	189	371	4,133,816	−842	−1302	−622
**Shanxi**	14,618,624	388	297	557	17,064,195	−2524	−3896	−1866
Taiyuan	2,189,596	−71	−113	−52	2,733,849	−259	−402	−191
Yuncheng	1,267,638	93	70	139	1,418,093	−334	−518	−247
**Inner Mongolia**	7,592,241	143	109	207	8,904,686	−775	−1205	−562
Hohhot	1,420,636	38	29	56	1,839,086	−105	−160	−77
Chifeng	1,025,914	43	32	64	1,079,600	−119	−183	−88
**Northeast China**	48,883,249	−979	−2062	−252	54,959,830	−1444	−2828	−549
**Liaoning**	21,903,437	662	506	953	24,758,787	−1320	−2119	−911
Shenyang	4,824,026	−35	−62	−24	5,723,392	−355	−549	−262
Dalian	3,464,468	−195	−305	−143	4,202,571	−80	−130	−58
**Jilin (province)**	11,742,940	−753	−1173	−554	12,803,860	−261	−603	−15
Changchun	3,591,105	−430	−663	−319	4,072,416	−109	−173	−79
Jilin (city)	2,390,369	−153	−238	−112	2,535,946	−121	−192	−88
**Heilongjiang**	15,236,871	−888	−1395	−650	17,397,183	137	−106	377
Harbin	4,995,380	−93	−157	−66	6,036,118	−123	−203	−88
Qiqihar	1,778,088	−87	−136	−64	1,898,996	4	3	4
**East China**	127,914,533	8767	6668	12,769	146,544,502	−12,277	−19,381	−8823
**Shanghai**	7,135,130	416	319	595	11,819,133	−909	−1410	−670
**Jiangsu**	23,614,032	1677	1278	2431	25,577,172	−2105	−3302	−1544
Nanjing	2,537,274	221	168	321	3,585,265	−510	−792	−376
Suzhou	2,284,195	184	141	264	2,776,148	−69	−115	−49
**Zhejiang**	12,888,906	696	535	993	16,324,765	−534	−876	−373
Hangzhou	1,290,796	147	112	214	2,001,079	−105	−167	−77
Wenzhou	3,098,810	97	76	135	3,893,660	−121	−195	−87
**Anhui**	18,024,866	1624	1230	2386	18,470,001	−2460	−3826	−1812
Hefei	2,743,056	627	472	932	2,593,970	−602	−934	−444
Fuyang	2,792,072	51	40	68	2,686,350	−234	−367	−172
**Fujian**	11,572,315	384	298	537	13,773,340	161	33	307
Fuzhou	2,448,391	−116	−188	−84	2,743,562	52	41	72
Quanzhou	3,118,399	291	220	428	3,700,398	132	101	193
**Jiangxi**	9,080,980	785	597	1145	10,589,480	−376	−605	−271
Nanchang	1,698,167	194	148	281	2,099,653	−122	−194	−89
Ganzhou	1,298,164	99	75	146	1,508,520	−27	−44	−19
**Shandong**	30,885,628	2894	2186	4278	33,993,997	−5362	−8306	−3958
Jinan	3,464,719	360	271	537	3,959,993	−907	−1403	−670
Qingdao	3,197,865	−92	−153	−65	3,858,206	−181	−288	−132
**Taiwan**	14,712,678	290	225	405	15,996,613	−693	−1089	−501
Taipei	1,900,784	−64	−99	−47	2,066,701	−91	−139	−67
New Taipei	2,973,444	−39	−64	−28	3,467,330	−153	−235	−113
**Central China**	160,744,566	2562	1924	3704	63,188,415	−7234	−11,284	−5305
**Henan**	27,355,634	1868	1420	2724	28,866,333	−4267	−6632	−3144
Zhengzhou	2,799,176	350	264	519	3,627,029	−557	−863	−411
Nanyang	2,282,542	−24	−45	−16	2,450,090	−198	−311	−145
**Hubei**	118,017,596	653	501	935	17,023,172	−1821	−2855	−1323
Wuhan	4,837,647	385	291	568	6,008,901	−956	−1477	−707
Huanggang	1,309,569	152	114	226	1,212,303	−117	−183	−86
**Hunan**	15,371,336	41	3	45	17,298,910	−1146	−1797	−837
Changsha	2,829,810	102	80	141	3,488,281	−304	−476	−224
Hengyang	1,622,018	101	77	148	1,797,319	−193	−299	−142
**South China**	40,192,320	−1381	−2311	−937	47,420,822	−295	−1127	358
**Guangdong**	27,684,031	−766	−1280	−543	33,402,262	−70	−624	393
Guangzhou	4,132,955	−245	−387	−179	4,284,891	−130	−206	−94
Shenzhen	4,089,798	−262	−413	−192	6,837,641	−334	−522	−245
**Guangxi**	6,792,098	−236	−381	−170	7,028,549	−25	−169	92
Nanning	1,630,976	7	6	7	1,524,833	−42	−70	−30
Yulin	813,699	−115	−178	−84	814,167	78	59	115
**Hainan**	1,822,040	81	62	115	2,367,704	−73	−121	−49
Haikou	857,405	88	67	129	1,230,207	−32	−52	−23
Sanya	137,743	28	21	41	198,897	−13	−21	−10
**Hong Kong**	3,740,298	−463	−715	−343	4,418,702	−121	−203	−77
**Macao**	153,853	3	2	4	203,606	−5	−10	−2
**Southwest China**	30,983,255	−609	−1451	−41	34,097,203	−2196	−3517	−1550
**Chongqing**	6,289,639	−382	−610	−278	6,419,435	−259	−416	−187
**Sichuan**	12,463,490	−612	−1009	−437	13,691,385	−555	−944	−357
Chengdu	1,827,642	145	112	204	3,172,810	−42	−79	−28
Nanchong	1,019,960	−99	−156	−72	1,163,448	−31	−52	−22
**Guizhou**	5,535,802	−142	−232	−102	6,184,507	−603	−948	−435
Guiyang	1,712,369	−49	−79	−35	2,172,016	−139	−219	−102
Bijie	678,063	41	31	60	756,111	−150	−232	−111
**Yunnan**	6,504,222	518	392	763	7,569,020	−775	−1203	−568
Kunming	2,854,477	233	177	343	3,366,233	−435	−669	−322
Qujing	814,286	103	77	152	905,324	−125	−194	−93
**Tibet**	190,103	9	7	14	232,857	−4	−5	−3
Lhasa	152,756	8	6	12	188,974	−2	−4	−2
Shigatse	13,998	0	0	1	16,754	0	0	0
**Northwest China**	24,738,979	−209	−525	1	29,374,686	−3134	−4894	−2281
**Shaanxi**	9,573,219	−50	−108	−30	10,732,585	−1579	−2451	−1165
Xi'an	2,570,049	8	4	8	3,185,587	−475	−737	−350
Weinan	1,658,703	79	60	116	1,749,916	−328	−507	−242
**Gansu**	5,646,554	−131	−211	−95	6,221,693	−719	−1112	−531
Lanzhou	1,891,964	−45	−71	−33	2,212,651	−122	−189	−91
Tianshui	750,997	−72	−111	−53	802,819	−217	−337	−160
**Qinghai**	1,311,369	176	132	262	1,642,268	−254	−391	−188
Xining	973,784	150	113	224	1,238,135	−219	−336	−162
Haidong	237,304	28	21	42	258,365	−30	−47	−22
**Ningxia**	1,931,743	20	16	26	2,499,877	−303	−469	−224
Yinchuan	961,495	35	27	52	1,375,652	−169	−260	−125
Wuzhong	316,252	−1	−3	−1	376,591	−40	−62	−29
**Xinjiang**	6,276,094	−223	−353	−163	8,278,262	−279	−471	−173
Urumchi	2,175,739	−98	−153	−72	3,156,405	−208	−322	−154
Kashgar	710,234	35	26	52	896,757	−7	−12	−5

**Fig. 4:**
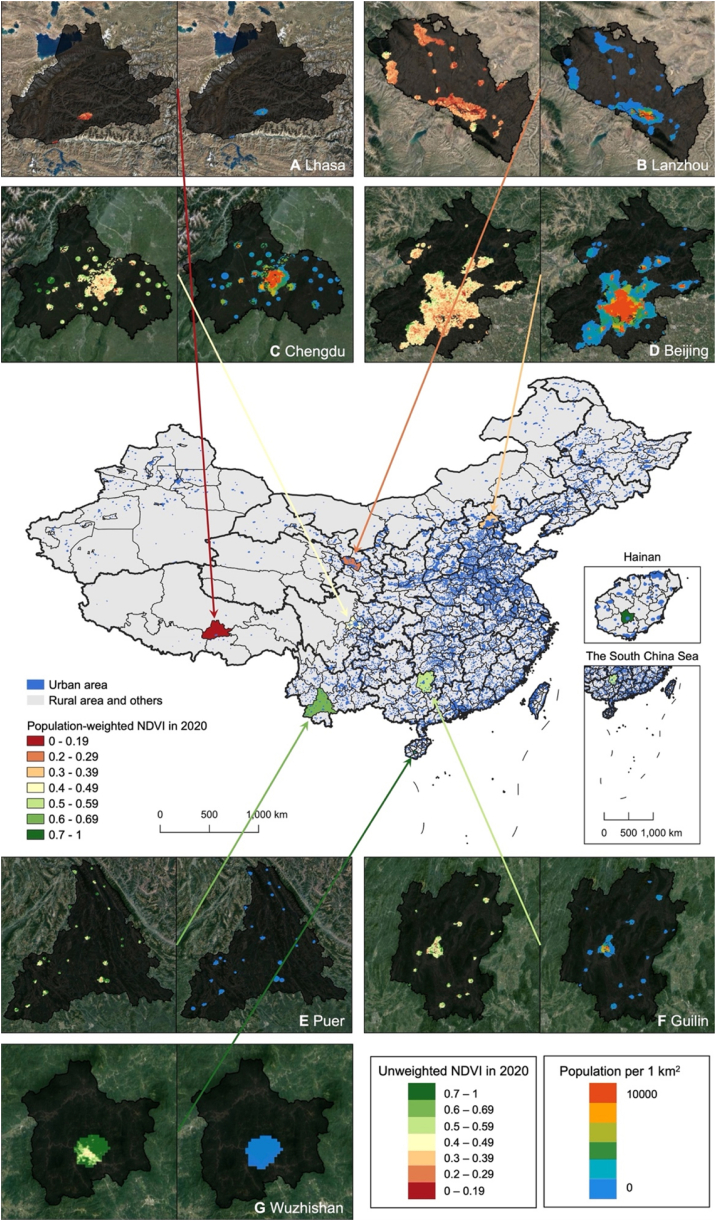
**Greenness exposure and population in seven sample cities**.

Fig. 4 shows the geospatial population density and population-weighted NDVI of seven selected cities with varying levels of greenness. The study found that the distribution of green space and population density was not uniform across all cities. The cities included Lhasa in Tibet, Lanzhou in Gansu, Beijing, Chengdu in Sichuan, Guilin in Guangxi, Puer in Yunnan, and Wuzhishan in Hainan, selected for their varying levels of greenness. In Lanzhou, Beijing, Chengdu, and Guilin, there was a noticeable difference in population density between the city center and surrounding areas, while the distribution of green space between the inner and outer city was less apparent. These findings indicate that different cities have unique distributions of green space and population density, highlighting the importance of considering these differences when analyzing their relationship. Uncertainty analysis indicates that the most significant source of uncertainty across all scenarios is the variation (or difference), followed by the population, and the ERF (Appendix p9, Figure S2).

When utilizing the unweighted NDVI to assess the actual greenness change, the estimated number of additional deaths decreases by 27% for the period 2000–2010 and averted deaths decreases by 7% for 2010–2020. Conversely, in counterfactual scenarios, the estimated number of preventable deaths shows an increase of 1% and 9%, respectively (Appendix p29, Table S4).

Employing the summer mean NDVI might reduce the estimated deaths. From 2000 to 2010, the sensitivity analysis shows that NDVI changes prevented 24,499 deaths, contrasting with the main analysis (−346%). In other scenarios, the summer NDVI estimates fewer averted deaths than the annual NDVI, with reductions between 13% and 37% (Appendix p29, Table S4).

Furthermore, the impact of varying RR is also assessed. For the actual green change, the estimates range from an 80% reduction to a 76% increase; for counterfactual scenarios, estimates vary widely, from a 95% increase to a 76% decrease (Appendix p30, Table S5).

## Discussion

Our study investigated the association between population-weighted NDVI and averted deaths in China from 2000 to 2020. The study results showed that an overall increase in population-weighted NDVI was linked to a reduction in averted deaths annually. Specifically, there was an increase of 9951 deaths annually for the NDVI decrease from 2000 to 2010, and a decrease of 37,653 deaths annually for the NDVI increase from 2010 to 2020, with the highest averted deaths observed in northern regions of the country. In addition, achieving target NDVI levels in 2000 might have averted 110,976 deaths and 118,330 deaths in 2010. This finding indicates that increasing greenness in Chinese cities can significantly affect health outcomes. The discussion compares the findings with prior studies and examines the causal mechanisms and implications of the study. The study's strengths and limitations are also discussed, and future research directions are identified.

The study found that an overall increase in population-weighted NDVI was associated with reduced deaths annually. The finding is particularly relevant as urbanization in China often involves expanding cities into peri-urban areas, leading to different patterns of urbanization and green space utilization compared to developed countries. This finding has important implications for urban planning and public health policies in China.

The study fills an evidence gap of green space trends and health impacts in China, which is the most populous country. A study in Europe found that meeting the WHO recommendation of access to green space for 2015 could prevent 42,968 deaths annually, representing 2.3% of total natural-cause mortality.^18^ China has different patterns of urbanization and green space utilization from these countries. Additionally, population density and socioeconomic factors are different between country to country. In some places, inner cities are less desirable, and suburbs are located at a driving distance from the cities, typically having more green spaces and socio-economic resources. In contrast, in Chinese cities, the inner areas are densely populated with favorable access to facilities, transportation, and healthcare, while peri-urban areas tend to be less densely populated with more access to green spaces.

In the uncertainty analysis, the change (or difference) in NDVI and the urban population are the most important sources of uncertainty, while ERF had little effect on mortality estimates. In the sensitivity analysis, using summer mean NDVI might lead to an underestimation of averted or preventable deaths. This could be because summer, being the peak of vegetation growth, shows less variation in NDVI compared to the annual average.

Our study has important implications for public health and urban planning policies in China. Green space plays a critical role in mitigating the effects of climate change by reducing the amount of heat absorbed by buildings and other infrastructure, improving air quality, reducing energy consumption, and increasing carbon sequestration.^19^ The study findings highlight the need to design and maintain green spaces in ways that promote health and well-being for all residents. China has implemented several policies and initiatives to increase the amount of green space in urban areas. These initiatives aim to increase the country's forest coverage to 24.1 percent by 2025 and create more livable, sustainable cities by promoting the creation of green spaces and reducing carbon emissions.^20^

The study has several strengths, including the comprehensive coverage of a large dataset of 390 urban cities with a total population of nearly 500 million individuals. The study utilized remote sensing data, specifically the MODIS sensor, to analyze the greenness trend across urban areas in China. The temporal coverage of the study provides evidence that changes in urban NDVI between 2000 and 2020 have been linked to a reduction of 27,702 deaths annually, highlighting the importance of greenness for population health in urban areas.

However, the study has limitations. Our study utilizes an ecological design with aggregated city-level data, which limits our ability to adjust for individual-level confounding factors. Traditional methods for confounder adjustment require detailed individual or subgroup data, which were not available for all cities and time periods covered in this study. The study relies on the NDVI as a proxy for green space coverage, which may not account for variations in the quality, quantity, or accessibility of green spaces. Additionally, we excluded water bodies in our calculations, as exposure to blue spaces may reduce the risk of death, which could lead to an overestimation of the mortality burden attributed to the lack of greenness. In this study, we employed a simple linear regression model to analyze the trends in urban greenness from 2000 to 2020. While intuitive, a simple linear model is based on the assumption of stability in time series data, which may be overly simplified in the context of rapid urbanization and climate change. Although our research reveals the general trend of greenness, structural changes or non-linear trend variations that may exist in reality could lead to biases in model estimation. Future studies could consider using better model fit time series analysis methods to interpret the dynamic changes in urban greenness. Finally, using relative risk estimates from studies conducted in other countries may not fully capture China's specific environmental, social, and demographic contexts. Factors like urban planning policies, cultural practices, and baseline health status can influence the greenness–mortality relationship differently. Despite adjustments in the original studies, unmeasured or inadequately adjusted confounders may still bias the RR—for example, areas with more green space might also have better air quality or lower noise pollution, which independently reduce mortality risk. Therefore, we emphasize the importance of conducting large-scale cohort studies within China to establish context-specific exposure–response relationships that account for local confounders and effect modifiers.

Further research using local data and context-specific exposure–response relationships is necessary to provide more accurate estimates and inform evidence-based policy decisions.

### Conclusion

Our study found that the decrease in urban NDVI from 2000 to 2010 could have led to 9951 additional deaths annually, while the increase in NDVI from 2010 to 2020 could have averted an estimated 37,653 deaths annually. If the NDVI were increased to the target value in 2000 and 2010, the number of deaths would be reduced by 110,976 and 118,330, respectively. The highest averted deaths were observed in the northern regions of the country compared to the southern regions. The study suggests that greenness could serve as an effective protective measure for population health, considering the ongoing impacts of climate change and urbanization. Sustained green space growth in urban areas offers significant health and climate adaptation benefits, but its effectiveness may be threatened by climate hazards such as heatwaves. Heat not only poses direct health risks but can also degrade green spaces, creating a double burden of disease. It is crucial to ensure that urban green spaces are resilient to such climate stressors for continue health benefits. Further research should also focus on determining the optimal amount of green space and a broader range of health outcomes. This will explain cause-specific averted mortality by green space changes. Our research is an initial step to providing insights for developing urban planning and public health strategies that intertwined the challenges of climate change, urbanization, and health.

## Contributors

John S. Ji and Wenjia Cai conceived the study. John S. Ji, Hui Miao, Zheng Tao collected, pre-processed and validated the underlying data. Hui Miao and Zheng Tao conducted statistical analyses. John S. Ji led the writing processes. Tom Cole-Hunter, Xuecao Li, David Rojas-Rueda, and Wenjia Cai contributed to resources and finding interpretation. Authors approved the final version of the manuscript. John S. Ji had final responsibility for the decision to submit for publication.

## Data sharing statement

The data used in this study are accessible on platforms with restricted access. NDVI and population data are available on the Google Earth Engine. The urban boundary data can be found at https://earthdata.nasa.gov/data/catalog/sedac-ciesin-sedac-grumpv1-stlmnt01-1.01. Data on national mortality rates are derived from the National Population Census. Coding is made available in GitHub and updated when necessary (https://github.com/johnjiresearchlab).

## Editor note

The Lancet Group takes a neutral position with respect to territorial claims in published maps and institutional affiliations.

## Declaration of interests

We declare no competing interests.
