# Supplementary material for: Greenness and averted mortality in 390 cities in China (2000–2020)
Source: Lancet Reg Health West Pac. 2025 Jan 16;54:101283. doi: 10.1016/j.lanwpc.2024.101283 (PMC11786093; doi:10.1016/j.lanwpc.2024.101283)
Supplement: Supplementary appendix [file mmc1.docx]

**Supplementary appendix**

Supplementary appendix

A. Definition of urban areas and cities **3**

B. Population and mortality**3**

C. Exposure to greenness**4**

D. Health impact assessment **4**

E. Counterfactual scenarios of green exposure**4**

F. Uncertainty analysis **6**

G. Sensitivity analysis **7**

H. Figures and tables **7**

References **31**

1. **Definition of urban areas and cities**

We obtained the list of Chinese cities from the Ministry of Civil Affairs of the People's Republic of China (1). China has a four-tier city system based on specific administrative levels: provincial-level municipalities directly under the central government, prefecture-level cities, county-level cities, and towns. Since county-level cities are part of prefecture-level cities and are geographically located within them, to avoid double counting, we selected all prefecture-level administrative units (including prefecture-level cities, regions, autonomous prefectures, and leagues) and 6 provincial-level units (including municipalities and special administrative regions) as the study areas (Table S1).

The urban areas within each city were delineated using extent polygons from the Global Rural-Urban Mapping Project (GRUMP) (Figure S1) (2,3). GRUMP delineates urban areas starting from global nighttime lights data, which are transformed into polygons representing urban extents, encompassing all urban areas with populations of 1,000 or more.

1. **Population and mortality**

We collected population data from the Gridded Population of World Version 4 (GPWv4), Revision 11 (4). GPWv4 provided information on the distribution of the global human population across 30 arc-second grid cells (roughly equivalent to 1km) for the years 2000, 2005, 2010, 2015, and 2020. To calculate the total population within the urban areas of each city, we summed the populations of the relevant grid cells. We also used national census statistics from 2000, 2010, and 2020 to determine the population aged 20 and above, based on age-specific population proportions (Table S1).

Since province-level mortality rates data was for the whole population, we used national-level mortality rates in 2000, and 2010 for population aged 20 and above, based on national annual statistics. Our study seeks to provide a comprehensive description of the relationship between population density, age demographics, and mortality rates in urban areas.

1. **Exposure to greenness**

**1) NDVI**

We utilized NDVI images from the Moderate Resolution Imaging Spectroradiometer (MODIS) sensor with a resolution of 250 meters to estimate green exposure. We obtained all MOD13Q1 images from 2000, 2005, 2010, 2015, and 2020 and calculated the median NDVI value on a pixel basis to obtain an NDVI median layer for each year, excluding potential outliers. We pre-processed the selected images, excluding water pixels.

**2) Population-weighted NDVI**

We calculated population-weighted NDVI for each city and province. This involved weighting the NDVI values for each pixel by the corresponding population density and then averaging across all pixels to obtain an overall population-weighted NDVI value for the city or province. The equation used to calculate the population-weighted NDVI value for each city and province is:

$$population-weighted NDVI=\frac{\sum_{i=1}^{n} (NDVI_{i} \times Pop_{i})}{\sum_{i=1}^{n} Pop_{i}}$$

where $n$ is the number of pixels in an urban area with valid NDVI data in a city or a province.

**3)** **Temporal trends**

we employed a simple linear regression model to examine temporal trends. Specifically, we set the number of preventable deaths as the dependent variable, and time (year) as the independent variable. The model is expressed as follows:

Adverted Deaths=β0 + β1 × Year + ϵ

In this model, β0 represents the intercept, β1 is the slope coefficient for time, and ϵ is the error term. We conducted a t-test on the slope coefficient β1 to determine if there is a significant impact of time on the number of preventable deaths. If the p-value exceeds the level of significance (0.05), we consider that there is no significant change over time.

1. **Health impact assessment**

We conducted a quantitative health impact assessment on 390 cities to estimate the effect of greenness on mortality among the population aged 20 years and above in China. Firstly, we estimated the deaths attributable to changes in greenness over a 20-year period in China. Secondly, based on previous health assessment frameworks (5,6), we evaluated the mortality impact due to the difference between actual exposure and counterfactual scenarios. The procedures are as follows:

1. The level of green exposure was estimated using the NDVI.

2. We obtained the exposure-response function (ERF) from existing literature (Table S2) to quantify the relationship between green exposure and mortality.

3. We calculated the changes in greenness between 2010 and 2000, and 2020 and 2010. Then, we estimated the baseline green exposure and calculated the differences between the actual exposure and counterfactual scenarios in 2000 and 2010.

4. We estimated the relative risk (RR) corresponding to these exposure changes or differences. Based on the exposure differences and RR, we calculated the attributable fraction (AF), which was used to estimate the mortality burden.

5. We conducted uncertainty analysis using 10,000 Monte Carlo simulations to obtain point estimates and confidence intervals (CIs).

6. We calculated the deaths for each city and aggregated the results across all cities.

We estimated the annual averted deaths attributable to NDVI changes (or difference). Mortality, $M$ is expressed as:

$$M=y_{0} \times Pop \times AF$$

where $y_{0}$ is the national-level annual all-cause mortality rate for population aged 20 and above in 2000 and 2010, $Pop$ is the population size and $AF$ is the attributable fraction of NDVI changes (or difference). $AF$ is calculated via the relative risk ($RR$), which represents the decrease of risk of mortality resulting from the NDVI changes (or difference). $AF$ is calculated as:

$$AF= \frac{RR-1}{RR}$$

A 0.1-unit increase of NDVI could reduce mortality risk by 4% (7) i.e. $RR_{0.1-unit-increase}=0.96 (95\% CI 0.94-0.97)$. The $RR$ of NDVI change is calculated as:

$$RR_{change}={RR_{0.1-unit-increase}}^{change/0.1}$$

where $change$ is NDVI in 2010 subtracted by that in 2000, or NDVI in 2020 subtracted by that in 2010.

Similarly, we calculated the RR for the ‘difference’ between the counterfactual exposure and the actual exposure level:

$$RR_{difference}=exp(\left( \frac{\ln\left( RR_{0.1-unit-increase} \right)}{0.1} \right)*\left( difference \right))$$

where $difference$ is the difference between the actual NDVI and the counterfactual target.

1. **Counterfactual scenarios of green exposure**

Counterfactual scenarios of green exposure are established to estimate the number of deaths in health impact assessments. We set the target level of greenness for the counterfactual exposure as the 75th percentile of NDVI at the city level. This standard is chosen under the assumption that 75% of cities are exposed to NDVI levels lower than the counterfactual level.

1. **Uncertainty analysis**

Uncertainty analyses were conducted on 7 regions in China to assess the impact of uncertainty distribution estimates of NDVI change (or difference), population, and ERF (Figure S2) on the mortality. Subsequently, we constructed an uncertainty distribution and obtained point estimates and 95% CI using 10,000 Monte Carlo simulations for the final estimates (Table S3).

We propagated the uncertainty of each parameter to the final results using Monte Carlo simulations. Initially, we estimated the distribution of the variables and calculated the mean and standard deviation for each variable. The 95% CIs in our final estimations were determined using the 2.5th and 97.5th percentiles from the Monte Carlo samples.

As the ERFs were derived from the meta-analysis, we assumed that the ERF estimates were normally distributed on a logarithmic scale. For each ERF, we used its 95% CI to compute the standard deviation in log (ERF) as follows:

𝒔𝒅(𝒍𝒐𝒈(𝑬𝑹𝑭)) = (𝒍𝒐𝒈(𝟗𝟓%𝑪𝑰(𝑬𝑹𝑭)𝒍𝒐𝒘𝒆𝒓 / 𝟗𝟓%𝑪𝑰(𝑬𝑹𝑭)𝒖𝒑𝒑𝒆𝒓)) /𝟐𝜱−𝟏(𝟎.𝟗𝟕𝟓)

where Φ−1(0.975) ≈ 1.96 is the 97.5th percentile of the standard normal distribution. We simulated values for log (ERF) using a normal distribution with mean the logarithm of the ERF point estimate and the standard deviation shown above. The simulated values for log (ERF) were exponentiated to get the simulated values for EFR.

1. **Sensitivity analysis**

We performed sensitivity analyses to assess the effect of changes in model variables on the final mortality estimates. We tested the effects of using different ERFs, as well as the effects of unweighted NDVI and the mean NDVI from June to August (i.e. the greenest period of the year) on mortality estimates (Table S4 and Table S5).

1. **Figures and tables**


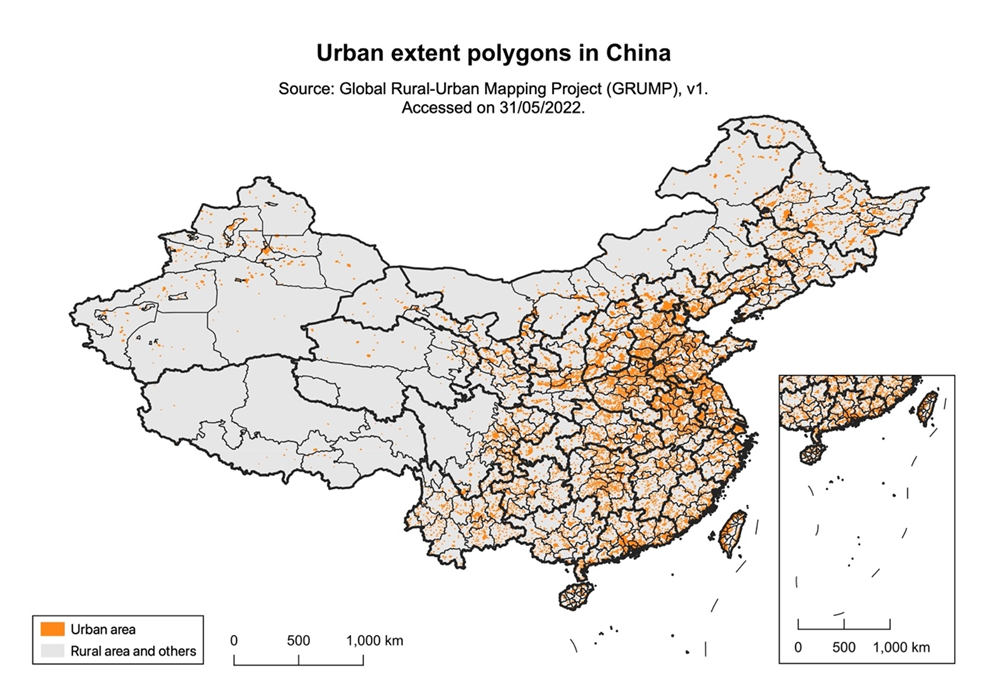


**Figure S1. Urban extent polygons in China.**


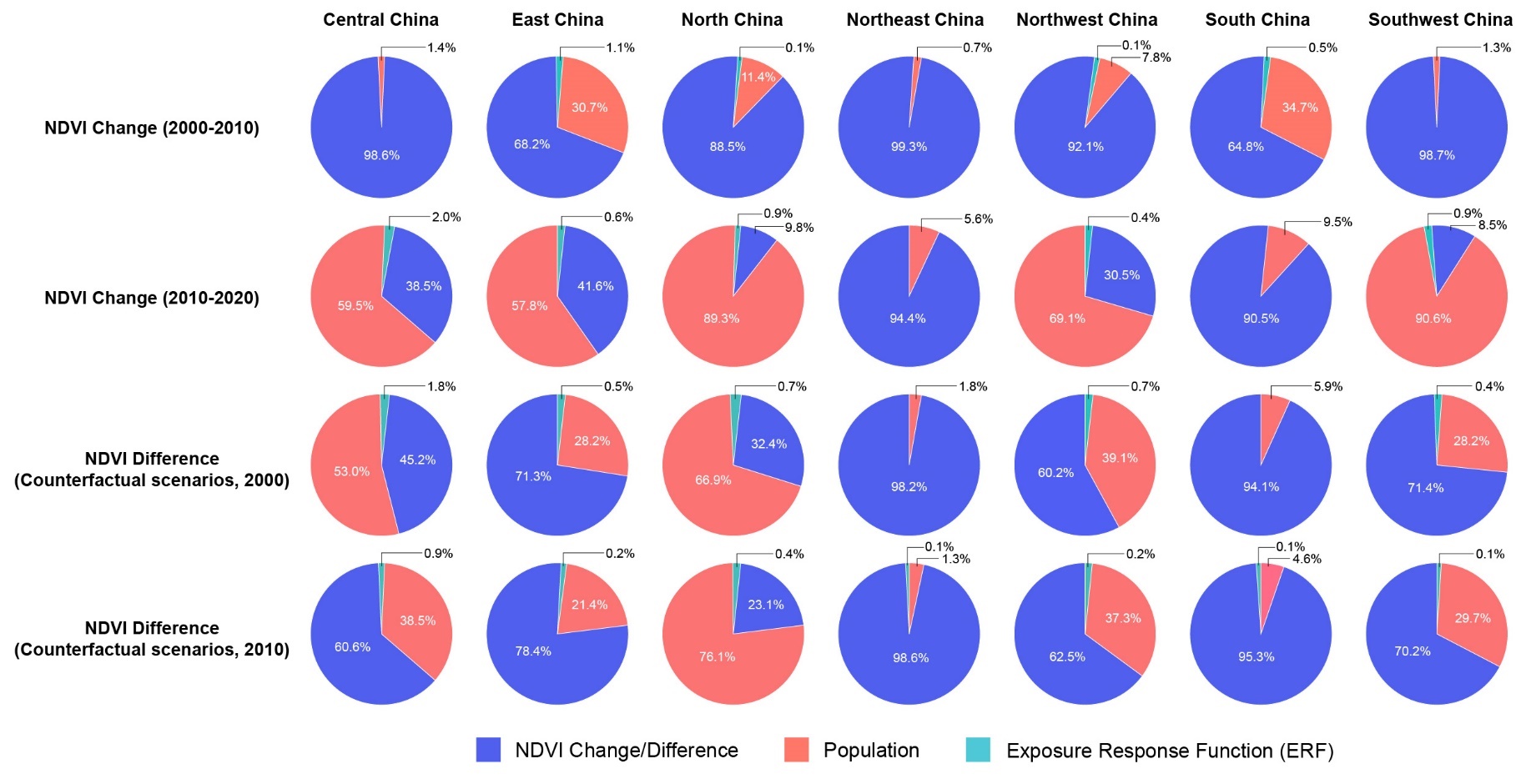


**Figure S2. Contribution of the variance of different variables in the uncertainty analysis.**

**Table S1. Urban population in China from 2000 to 2020**

| **Province** | **City** | | **Population in urban area** | | | | |
| --- | --- | --- | --- | --- | --- | --- | --- |
|  |  |  | **2000** | **2005** | **2010** | **2015** | **2020** |
| Beijing | Beijing | | 15,202,756 | 18,305,930 | 20,929,900 | 25,806,415 | 31,016,141 |
| Hebei Province | Baoding | | 4,643,735 | 4,801,889 | 4,957,389 | 5,113,533 | 5,262,554 |
| Hebei Province | Cangzhou | | 2,314,135 | 2,398,983 | 2,486,458 | 2,574,430 | 2,667,416 |
| Hebei Province | Chengde | | 905,749 | 925,852 | 946,405 | 967,418 | 988,902 |
| Hebei Province | Handan | | 4,851,126 | 5,073,006 | 5,291,446 | 5,522,111 | 5,756,019 |
| Hebei Province | Hengshui | | 1,426,971 | 1,458,418 | 1,490,561 | 1,504,285 | 1,528,825 |
| Hebei Province | Langfang City | | 1,685,743 | 1,798,182 | 1,915,910 | 2,030,671 | 2,146,113 |
| Hebei Province | Qinhuangdao | | 1,869,420 | 1,944,946 | 2,024,190 | 2,110,990 | 2,195,622 |
| Hebei Province | Shijiazhuang City | | 5,905,339 | 6,193,060 | 6,487,869 | 6,657,214 | 7,128,291 |
| Hebei Province | Tangshan | | 4,423,317 | 4,589,415 | 4,762,734 | 4,949,885 | 5,136,263 |
| Hebei Province | Xingtai City | | 2,976,851 | 3,077,937 | 3,134,991 | 3,207,200 | 3,388,474 |
| Hebei Province | Zhangjiakou | | 2,248,863 | 2,290,057 | 2,332,010 | 2,374,529 | 2,418,163 |
| Inner Mongolia Autonomous Region | Alxa League | | 7,176 | 7,851 | 8,597 | 9,052 | 10,195 |
| Inner Mongolia Autonomous Region | Baotou | | 2,287,951 | 2,481,309 | 2,690,495 | 2,896,387 | 3,152,891 |
| Inner Mongolia Autonomous Region | Bayannur City | | 586,795 | 584,568 | 582,350 | 580,141 | 577,939 |
| Inner Mongolia Autonomous Region | Chifeng | | 1,432,681 | 1,417,327 | 1,402,134 | 1,386,991 | 1,368,161 |
| Inner Mongolia Autonomous Region | Hohhot | | 1,908,686 | 2,089,093 | 2,286,553 | 2,502,424 | 2,739,227 |
| Inner Mongolia Autonomous Region | Hulunbeir | | 1,255,242 | 1,228,227 | 1,201,420 | 1,157,515 | 1,128,684 |
| Inner Mongolia Autonomous Region | Ordos City | | 516,840 | 610,287 | 721,039 | 850,571 | 1,005,342 |
| Inner Mongolia Autonomous Region | Tongliao | | 877,876 | 892,572 | 907,364 | 892,188 | 899,136 |
| Inner Mongolia Autonomous Region | Wuhai City | | 361,014 | 402,731 | 449,280 | 494,589 | 558,808 |
| Inner Mongolia Autonomous Region | Wulanchabu | | 664,705 | 643,937 | 623,822 | 604,339 | 585,200 |
| Inner Mongolia Autonomous Region | Xilin Gol League | | 198,752 | 204,068 | 209,526 | 212,838 | 210,936 |
| Inner Mongolia Autonomous Region | Xing'an League | | 334,072 | 336,623 | 339,195 | 337,460 | 340,533 |
| Shanxi Province | Changzhi City | | 1,796,181 | 1,851,293 | 1,908,097 | 1,964,815 | 2,019,708 |
| Shanxi Province | Datong | | 2,242,047 | 2,357,360 | 2,478,624 | 2,606,145 | 2,740,243 |
| Shanxi Province | Jincheng City | | 1,460,831 | 1,500,238 | 1,540,585 | 1,581,735 | 1,621,087 |
| Shanxi Province | Jinzhong | | 1,877,204 | 1,949,026 | 2,023,646 | 2,091,323 | 2,173,497 |
| Shanxi Province | Linfen City | | 2,651,934 | 2,716,030 | 2,895,682 | 2,899,370 | 2,918,068 |
| Shanxi Province | Luliang City | | 2,011,676 | 2,111,737 | 2,216,809 | 2,327,142 | 2,442,069 |
| Shanxi Province | Shuozhou | | 797,694 | 866,819 | 941,925 | 1,023,529 | 1,111,484 |
| Shanxi Province | Taiyuan City | | 3,708,608 | 4,110,451 | 3,936,336 | 4,186,412 | 5,072,811 |
| Shanxi Province | Xinzhou | | 1,173,068 | 1,198,645 | 1,224,793 | 1,251,527 | 1,268,892 |
| Shanxi Province | Yangquan City | | 1,076,802 | 1,116,409 | 1,157,472 | 1,200,046 | 1,244,186 |
| Shanxi Province | Yuncheng | | 1,837,308 | 1,917,796 | 1,854,386 | 1,926,121 | 1,911,947 |
| Tianjin | Tianjin | | 9,113,315 | 10,434,910 | 11,941,546 | 13,694,436 | 15,691,815 |
| Heilongjiang Province | Daqing City | | 1,762,678 | 1,870,150 | 1,985,530 | 2,073,340 | 2,221,385 |
| Heilongjiang Province | Greater Khingan Mountains | | 39,186 | 39,407 | 39,629 | 39,834 | 40,041 |
| Heilongjiang Province | Harbin City | | 6,706,454 | 7,128,680 | 7,577,493 | 8,001,381 | 8,480,186 |
| Heilongjiang Province | Hegang City | | 967,446 | 949,871 | 932,632 | 915,703 | 899,014 |
| Heilongjiang Province | Heihe City | | 606,407 | 606,768 | 607,135 | 605,779 | 588,216 |
| Heilongjiang Province | Jiamusi City | | 1,358,174 | 1,412,242 | 1,468,491 | 1,526,856 | 1,585,235 |
| Heilongjiang Province | Jixi City | | 1,464,861 | 1,432,941 | 1,401,715 | 1,371,172 | 1,341,211 |
| Heilongjiang Province | Mudanjiang City | | 1,322,381 | 1,350,222 | 1,378,650 | 1,407,328 | 1,427,665 |
| Heilongjiang Province | Qiqihar City | | 2,519,566 | 2,507,586 | 2,495,465 | 2,483,087 | 2,466,932 |
| Heilongjiang Province | Qitaihe | | 725,043 | 774,474 | 819,183 | 880,277 | 938,491 |
| Heilongjiang Province | Shuangyashan City | | 749,226 | 752,912 | 756,617 | 759,551 | 762,806 |
| Heilongjiang Province | Suihua City | | 1,813,267 | 1,880,161 | 1,949,601 | 2,003,769 | 2,050,696 |
| Heilongjiang Province | Yichun | | 805,839 | 772,445 | 740,437 | 709,757 | 680,351 |
| Jilin Province | Baicheng | | 1,036,963 | 1,038,623 | 1,040,288 | 925,589 | 982,500 |
| Jilin Province | Baishan City | | 771,165 | 766,409 | 761,683 | 756,987 | 752,321 |
| Jilin Province | Changchun City | | 4,976,007 | 5,160,347 | 5,318,106 | 5,298,795 | 5,600,951 |
| Jilin Province | Jilin City | | 3,359,292 | 3,332,104 | 3,305,132 | 3,274,946 | 3,241,196 |
| Jilin Province | Liaoyuan City | | 783,632 | 755,992 | 729,363 | 703,711 | 677,609 |
| Jilin Province | Siping City | | 1,452,904 | 1,473,505 | 1,494,408 | 1,479,206 | 1,474,888 |
| Jilin Province | Songyuan City | | 981,375 | 1,001,403 | 1,021,849 | 1,037,839 | 1,041,481 |
| Jilin Province | Tonghua City | | 1,264,161 | 1,268,627 | 1,273,111 | 1,273,897 | 1,271,722 |
| Jilin Province | Yanbian Korean Autonomous Prefecture | | 1,612,948 | 1,635,116 | 1,657,514 | 1,672,936 | 1,672,066 |
| Liaoning Province | Anshan City | | 2,825,473 | 2,849,308 | 2,871,570 | 2,876,241 | 2,909,251 |
| Liaoning Province | Benxi | | 1,097,417 | 1,146,086 | 1,196,938 | 1,249,952 | 1,305,402 |
| Liaoning Province | Chaoyang City | | 1,565,059 | 1,527,880 | 1,491,584 | 1,447,823 | 1,405,493 |
| Liaoning Province | Dalian | | 4,911,510 | 5,231,327 | 5,571,316 | 5,919,441 | 6,296,204 |
| Liaoning Province | Dandong | | 1,370,139 | 1,383,831 | 1,401,540 | 1,413,146 | 1,434,137 |
| Liaoning Province | Fushun City | | 1,778,402 | 1,730,659 | 1,684,286 | 1,637,816 | 1,593,876 |
| Liaoning Province | Fuxin City | | 1,165,064 | 1,143,199 | 1,121,747 | 1,100,317 | 1,077,698 |
| Liaoning Province | Huludao City | | 1,515,563 | 1,527,431 | 1,540,306 | 1,552,765 | 1,565,918 |
| Liaoning Province | Jinzhou City | | 1,855,546 | 1,870,377 | 1,885,395 | 1,898,779 | 1,914,129 |
| Liaoning Province | Liaoyang City | | 1,237,945 | 1,257,631 | 1,277,636 | 1,297,893 | 1,318,547 |
| Liaoning Province | Panjin | | 1,138,839 | 1,195,424 | 1,254,856 | 1,317,174 | 1,382,741 |
| Liaoning Province | Shenyang City | | 6,652,405 | 7,055,979 | 7,468,428 | 7,664,211 | 8,216,822 |
| Liaoning Province | Tieling City | | 1,246,565 | 1,223,346 | 1,200,578 | 1,174,104 | 1,150,231 |
| Liaoning Province | Yingkou | | 1,636,171 | 1,675,252 | 1,728,172 | 1,752,355 | 1,780,399 |
| Anhui Province | Anqing | | 1,516,658 | 1,481,270 | 1,409,144 | 1,352,829 | 1,417,449 |
| Anhui Province | Bengbu | | 1,755,048 | 1,719,667 | 1,679,211 | 1,584,635 | 1,580,047 |
| Anhui Province | Bozhou | | 1,897,462 | 1,854,482 | 1,804,169 | 1,692,700 | 1,663,245 |
| Anhui Province | Chizhou | | 231,101 | 232,025 | 232,890 | 233,408 | 235,053 |
| Anhui Province | Chuzhou | | 832,645 | 826,076 | 812,106 | 766,138 | 777,779 |
| Anhui Province | Fuyang City | | 3,745,071 | 3,628,209 | 3,505,979 | 3,051,867 | 3,139,240 |
| Anhui Province | Hefei | | 4,581,325 | 4,203,438 | 4,549,874 | 3,827,697 | 4,157,021 |
| Anhui Province | | Huaibei | 1,357,800 | 1,440,064 | 1,380,867 | 1,376,594 | 1,487,828 |
| Anhui Province | | Huainan City | 1,881,231 | 1,962,807 | 2,041,492 | 2,193,162 | 2,294,355 |
| Anhui Province | | Huangshan City | 342,548 | 342,366 | 340,587 | 337,836 | 339,534 |
| Anhui Province | | Lu'an City | 1,273,822 | 1,236,793 | 1,189,238 | 1,102,879 | 1,100,809 |
| Anhui Province | | Ma'anshan | 1,198,321 | 1,197,510 | 1,012,925 | 1,104,654 | 1,186,065 |
| Anhui Province | | Suzhou City | 2,149,037 | 2,119,425 | 2,080,686 | 1,906,402 | 1,946,159 |
| Anhui Province | | Tongling | 785,418 | 804,051 | 809,684 | 815,355 | 856,173 |
| Anhui Province | | Wuhu | 1,760,239 | 1,786,824 | 1,748,222 | 1,669,442 | 1,843,075 |
| Anhui Province | | Xuancheng | 591,165 | 577,054 | 557,250 | 540,775 | 529,928 |
| Fujian Province | | Fuzhou | 4,185,180 | 3,478,370 | 3,548,827 | 3,230,104 | 3,992,740 |
| Fujian Province | | Longyan City | 929,693 | 907,843 | 880,486 | 848,700 | 841,612 |
| Fujian Province | | Nanping | 817,622 | 792,417 | 744,253 | 617,310 | 719,514 |
| Fujian Province | | Ningde | 901,989 | 875,409 | 841,488 | 802,062 | 801,299 |
| Fujian Province | | Putian City | 1,567,246 | 1,583,163 | 1,590,184 | 1,581,048 | 1,620,662 |
| Fujian Province | | Quanzhou | 4,545,855 | 4,659,471 | 4,771,067 | 4,789,052 | 5,404,021 |
| Fujian Province | | Sanming City | 817,813 | 834,770 | 728,501 | 695,897 | 771,840 |
| Fujian Province | | Xiamen City | 1,694,484 | 2,229,489 | 2,768,468 | 3,641,472 | 4,936,090 |
| Fujian Province | | Zhangzhou | 2,056,365 | 2,050,928 | 1,878,499 | 1,944,275 | 2,055,999 |
| Jiangsu Province | | Changzhou City | 1,826,874 | 1,375,363 | 1,419,088 | 1,515,859 | 2,592,772 |
| Jiangsu Province | | Huaian City | 2,779,052 | 2,707,840 | 2,602,479 | 2,356,107 | 2,406,768 |
| Jiangsu Province | | Lianyungang | 2,614,131 | 2,566,312 | 2,494,609 | 2,349,921 | 2,356,762 |
| Jiangsu Province | | Nanjing | 4,284,538 | 4,610,028 | 4,502,718 | 4,762,576 | 7,314,771 |
| Jiangsu Province | | Nantong City | 2,686,770 | 2,553,934 | 2,256,596 | 1,866,270 | 2,293,065 |
| Jiangsu Province | | Suqian | 2,528,084 | 2,440,610 | 2,323,145 | 2,173,589 | 2,116,288 |
| Jiangsu Province | | Suzhou City | 4,304,925 | 4,090,179 | 3,900,293 | 3,897,791 | 5,770,947 |
| Jiangsu Province | | Taizhou | 1,861,408 | 1,819,195 | 1,605,275 | 1,346,955 | 1,535,361 |
| Jiangsu Province | | Wuxi | 2,878,932 | 2,689,763 | 2,421,790 | 2,880,238 | 4,009,642 |
| Jiangsu Province | | Xuzhou | 4,650,826 | 4,756,189 | 4,009,089 | 3,153,391 | 3,766,328 |
| Jiangsu Province | | Yancheng City | 3,399,965 | 3,236,811 | 3,000,666 | 2,607,110 | 2,661,643 |
| Jiangsu Province | | Yangzhou | 2,183,455 | 2,100,051 | 1,839,743 | 1,666,470 | 1,849,882 |
| Jiangsu Province | | Zhenjiang | 1,304,834 | 1,363,547 | 1,379,384 | 1,375,012 | 1,538,168 |
| Jiangxi Province | | Fuzhou | 927,075 | 973,673 | 1,022,614 | 1,073,567 | 1,128,000 |
| Jiangxi Province | | Ganzhou City | 1,904,214 | 2,022,499 | 2,063,842 | 1,884,327 | 2,332,213 |
| Jiangxi Province | | Ji'an City | 944,391 | 991,561 | 1,040,386 | 1,093,953 | 1,148,738 |
| Jiangxi Province | | Jingdezhen | 632,252 | 660,782 | 690,601 | 721,764 | 754,334 |
| Jiangxi Province | | Jiujiang City | 1,329,484 | 1,376,143 | 1,404,286 | 1,219,340 | 1,522,732 |
| Jiangxi Province | | Nanchang | 3,256,724 | 2,661,741 | 2,840,548 | 2,588,661 | 4,253,644 |
| Jiangxi Province | | Pingxiang | 775,288 | 712,794 | 830,499 | 797,652 | 738,940 |
| Jiangxi Province | | Shangrao | 1,556,033 | 1,632,673 | 1,687,805 | 1,733,651 | 1,883,071 |
| Jiangxi Province | | Xinyu City | 504,699 | 514,714 | 535,383 | 456,859 | 485,034 |
| Jiangxi Province | | Yichun City | 1,238,547 | 1,283,398 | 1,350,281 | 1,407,534 | 1,494,132 |
| Jiangxi Province | | Yingtan City | 408,872 | 427,951 | 447,921 | 468,202 | 490,046 |
| Shandong Province | | Binzhou | 1,456,497 | 1,494,200 | 1,531,075 | 1,537,248 | 1,599,944 |
| Shandong Province | | City Of Yantai | 3,411,601 | 3,494,766 | 3,560,943 | 3,639,349 | 3,716,762 |
| Shandong Province | | Dezhou | 2,339,795 | 2,399,962 | 2,447,441 | 2,434,170 | 2,501,861 |
| Shandong Province | | Dongying City | 1,177,193 | 1,253,887 | 1,331,452 | 1,392,706 | 1,497,772 |
| Shandong Province | | Heze City | 3,039,372 | 3,074,728 | 3,093,096 | 2,967,454 | 2,931,041 |
| Shandong Province | | Jinan City | 4,869,873 | 5,196,950 | 5,356,205 | 4,890,401 | 5,875,314 |
| Shandong Province | | Jining | 4,149,377 | 4,213,589 | 4,186,675 | 3,892,531 | 4,171,342 |
| Shandong Province | | Liaocheng | 2,036,776 | 2,106,244 | 2,171,106 | 2,151,506 | 2,176,461 |
| Shandong Province | | Linyi City | 4,090,429 | 3,796,399 | 3,558,043 | 3,359,151 | 3,461,271 |
| Shandong Province | | Qingdao | 4,874,125 | 5,056,012 | 5,165,954 | 5,895,641 | 6,251,113 |
| Shandong Province | | Rizhao City | 1,008,807 | 1,028,113 | 1,047,374 | 1,018,062 | 1,001,424 |
| Shandong Province | | Tai'an City | 2,104,339 | 2,135,859 | 2,124,549 | 2,038,118 | 2,173,674 |
| Shandong Province | | Weifang | 3,315,041 | 3,418,108 | 3,490,553 | 3,399,601 | 3,616,434 |
| Shandong Province | | Weihai | 1,555,002 | 1,614,497 | 1,677,538 | 1,741,891 | 1,802,972 |
| Shandong Province | | Zaozhuang | 1,661,910 | 1,703,164 | 1,701,658 | 1,658,449 | 1,707,989 |
| Shandong Province | | Zibo | 3,063,455 | 3,162,077 | 3,086,313 | 3,162,959 | 3,389,004 |
| Shanghai | | Shanghai | 12,589,644 | 14,136,321 | 14,896,848 | 19,975,297 | 29,767,106 |
| Taiwan Province | | Changhua County | 1,326,498 | 1,327,058 | 1,311,215 | 1,218,282 | 1,218,331 |
| Taiwan Province | | Chiayi City | 324,529 | 349,864 | 369,964 | 385,716 | 375,510 |
| Taiwan Province | | Chiayi County | 387,005 | 372,056 | 355,977 | 340,915 | 324,071 |
| Taiwan Province | | Hsinchu City | 383,410 | 409,036 | 435,508 | 463,663 | 484,834 |
| Taiwan Province | | Hsinchu County | 477,342 | 512,950 | 550,910 | 590,148 | 629,602 |
| Taiwan Province | | Hualien County | 232,389 | 240,358 | 238,215 | 227,332 | 216,248 |
| Taiwan Province | | Kaohsiung City | 2,121,369 | 2,575,919 | 2,320,836 | 1,828,262 | 2,379,759 |
| Taiwan Province | | Keelung | 615,627 | 619,798 | 619,535 | 620,976 | 596,234 |
| Taiwan Province | | Kinmen County | 32,505 | 32,583 | 32,435 | 32,534 | 33,971 |
| Taiwan Province | | Lianjiang County (Matsu) | 5,618 | 4,545 | 4,626 | 3,903 | 3,329 |
| Taiwan Province | | Miaosu County | 508,581 | 508,891 | 508,565 | 505,863 | 501,477 |
| Taiwan Province | | Nantou County | 305,386 | 299,440 | 289,759 | 282,128 | 277,067 |
| Taiwan Province | | New Taipei City | 3,907,577 | 4,054,713 | 4,229,024 | 4,405,216 | 4,576,086 |
| Taiwan Province | | Penghu County | 38,171 | 39,871 | 40,835 | 40,531 | 42,390 |
| Taiwan Province | | Pingtung County | 793,199 | 774,208 | 746,767 | 696,192 | 676,801 |
| Taiwan Province | | Taichung | 2,259,256 | 2,336,552 | 2,272,312 | 2,305,301 | 2,392,837 |
| Taiwan Province | | Tainan City | 1,431,446 | 1,294,112 | 1,299,789 | 1,216,786 | 1,243,772 |
| Taiwan Province | | Taipei City | 2,496,711 | 2,512,428 | 2,526,654 | 2,527,458 | 2,518,054 |
| Taiwan Province | | Taitung County | 131,762 | 126,493 | 127,167 | 124,050 | 126,759 |
| Taiwan Province | | Taoyuan City | 2,027,718 | 2,212,727 | 2,345,748 | 2,374,723 | 2,164,581 |
| Taiwan Province | | Yilan County | 372,110 | 360,891 | 364,688 | 349,662 | 341,454 |
| Taiwan Province | | Yunlin County | 516,621 | 488,158 | 458,999 | 417,714 | 385,119 |
| Zhejiang Province | | Hangzhou City | 2,847,556 | 2,817,013 | 2,738,628 | 3,287,554 | 5,521,620 |
| Zhejiang Province | | Huzhou | 894,610 | 863,662 | 849,697 | 868,501 | 1,029,137 |
| Zhejiang Province | | Jiaxing | 1,151,720 | 1,180,389 | 1,221,083 | 1,436,690 | 1,729,705 |
| Zhejiang Province | | Jinhua City | 2,189,167 | 2,251,884 | 2,190,383 | 2,121,828 | 2,769,889 |
| Zhejiang Province | | Lishui City | 767,426 | 710,503 | 715,933 | 658,676 | 674,241 |
| Zhejiang Province | | Ningbo | 2,937,097 | 2,795,065 | 2,942,474 | 3,415,763 | 5,093,984 |
| Zhejiang Province | | Quzhou City | 795,571 | 781,440 | 702,323 | 638,940 | 693,962 |
| Zhejiang Province | | Shaoxing | 2,108,087 | 2,023,107 | 1,901,667 | 2,001,381 | 2,451,120 |
| Zhejiang Province | | Taizhou | 2,710,977 | 2,535,471 | 2,494,552 | 2,684,744 | 3,108,827 |
| Zhejiang Province | | Wenzhou City | 5,476,940 | 4,678,001 | 5,161,821 | 6,256,405 | 7,206,275 |
| Zhejiang Province | | Zhoushan City | 549,600 | 560,083 | 593,138 | 636,304 | 685,842 |
| Henan Province | | Anyang City | 3,081,662 | 3,088,489 | 3,046,276 | 2,970,688 | 2,962,260 |
| Henan Province | | Hebi | 820,906 | 867,483 | 914,669 | 959,820 | 1,022,887 |
| Henan Province | | Jiaozuo City | 1,861,714 | 1,859,214 | 1,765,565 | 1,675,466 | 1,837,019 |
| Henan Province | | Jiyuan City | 291,938 | 303,194 | 313,382 | 317,130 | 336,842 |
| Henan Province | | Kaifeng | 2,176,908 | 2,197,909 | 2,202,229 | 2,188,263 | 2,166,132 |
| Henan Province | | Luohe City | 1,305,573 | 1,383,119 | 1,422,023 | 1,388,295 | 1,444,887 |
| Henan Province | | Luoyang City | 3,339,825 | 3,413,069 | 3,217,486 | 3,289,713 | 3,390,328 |
| Henan Province | | Nanyang City | 3,225,550 | 3,338,571 | 3,336,508 | 3,320,528 | 3,344,314 |
| Henan Province | | Pingdingshan City | 2,784,442 | 2,813,783 | 2,754,603 | 2,789,130 | 2,807,531 |
| Henan Province | | Puyang City | 2,074,176 | 2,116,117 | 2,152,655 | 2,144,206 | 2,061,246 |
| Henan Province | | Sanmenxia | 1,157,905 | 1,172,876 | 1,187,643 | 1,203,408 | 1,174,535 |
| Henan Province | | Shangqiu | 2,158,963 | 2,104,008 | 2,037,444 | 1,890,915 | 1,780,087 |
| Henan Province | | Xinxiang | 3,058,332 | 2,956,523 | 3,019,542 | 2,902,812 | 2,833,302 |
| Henan Province | | Xinyang City | 1,481,307 | 1,433,137 | 1,385,779 | 1,304,692 | 1,264,331 |
| Henan Province | | Xuchang | 1,837,307 | 1,869,880 | 1,840,087 | 1,762,446 | 1,729,788 |
| Henan Province | | Zhengzhou City | 4,772,779 | 4,504,533 | 4,803,977 | 6,003,097 | 7,537,108 |
| Henan Province | | Zhoukou City | 2,124,573 | 2,037,346 | 1,948,309 | 1,666,292 | 1,542,565 |
| Henan Province | | Zhumadian City | 1,717,348 | 1,691,734 | 1,632,331 | 1,479,832 | 1,486,735 |
| Hubei Province | | Enshi Tujia and Miao Autonomous Prefecture | 513,309 | 470,577 | 435,001 | 405,070 | 373,251 |
| Hubei Province | | Ezhou | 526,972 | 533,368 | 536,966 | 537,890 | 549,445 |
| Hubei Province | | Huanggang City | 1,778,709 | 1,658,968 | 1,530,521 | 1,326,642 | 1,318,945 |
| Hubei Province | | Huangshi City | 1,300,498 | 1,287,800 | 1,275,123 | 1,187,222 | 1,245,473 |
| Hubei Province | | Jingmen | 741,457 | 732,563 | 689,261 | 568,638 | 643,546 |
| Hubei Province | | Jingzhou | 2,157,662 | 2,030,226 | 1,642,089 | 1,421,258 | 1,644,551 |
| Hubei Province | | Qianjiang City | 409,281 | 381,689 | 344,665 | 301,668 | 287,699 |
| Hubei Province | | Shennongjia Forest Area | 157 | 147 | 137 | 128 | 120 |
| Hubei Province | | Shiyan City | 1,424,031 | 1,408,380 | 1,380,679 | 1,326,298 | 1,349,165 |
| Hubei Province | | Suizhou | 487,743 | 455,431 | 425,286 | 383,527 | 356,359 |
| Hubei Province | | Tianmen City | 326,857 | 304,829 | 279,939 | 239,833 | 242,579 |
| Hubei Province | | Wuhan | 7,210,518 | 7,725,150 | 7,572,058 | 8,128,864 | 9,699,338 |
| Hubei Province | | Xiangyang City | 2,144,637 | 2,113,669 | 1,959,760 | 1,794,703 | 1,857,743 |
| Hubei Province | | Xianning | 736,543 | 696,323 | 669,671 | 621,126 | 587,603 |
| Hubei Province | | Xiantao City | 300,367 | 280,219 | 245,115 | 214,383 | 199,696 |
| Hubei Province | | Xiaogan City | 1,937,888 | 1,902,973 | 1,811,956 | 1,754,933 | 1,680,036 |
| Hubei Province | | Yichang City | 1,680,587 | 1,469,445 | 1,405,027 | 1,386,622 | 1,492,911 |
| Hunan Province | | Changde City | 1,881,637 | 1,877,098 | 1,872,973 | 1,861,427 | 1,854,798 |
| Hunan Province | | Changsha City | 3,953,112 | 3,793,571 | 4,452,314 | 4,646,527 | 5,182,077 |
| Hunan Province | | Chenzhou | 918,422 | 1,043,991 | 867,995 | 860,652 | 976,409 |
| Hunan Province | | Hengyang | 2,311,844 | 2,360,967 | 2,266,768 | 2,453,899 | 2,507,675 |
| Hunan Province | | Huaihua City | 963,961 | 874,102 | 944,199 | 822,991 | 954,371 |
| Hunan Province | | Loudi | 1,359,236 | 1,359,630 | 1,354,606 | 1,144,638 | 1,291,207 |
| Hunan Province | | Shaoyang | 2,163,476 | 2,178,688 | 2,197,039 | 2,188,938 | 2,226,145 |
| Hunan Province | | Xiangtan City | 1,324,103 | 1,343,722 | 1,352,467 | 1,314,994 | 1,396,125 |
| Hunan Province | | Xiangxi Tujia and Miao Autonomous Prefecture | 369,388 | 388,963 | 401,868 | 399,712 | 420,381 |
| Hunan Province | | Yiyang City | 998,570 | 997,806 | 998,987 | 994,112 | 998,109 |
| Hunan Province | | Yongzhou | 1,362,828 | 1,340,296 | 1,319,453 | 1,273,209 | 1,260,057 |
| Hunan Province | | Yueyang City | 1,948,101 | 2,070,462 | 2,192,368 | 2,054,377 | 2,363,161 |
| Hunan Province | | Zhangjiajie City | 311,418 | 343,759 | 215,746 | 247,020 | 290,732 |
| Hunan Province | | Zhuzhou City | 1,586,670 | 1,646,423 | 1,683,962 | 1,721,497 | 1,838,947 |
| Guangdong Province | | Chaozhou | 1,101,343 | 1,061,514 | 983,103 | 902,997 | 1,081,371 |
| Guangdong Province | | Dongguan City | 6,171,166 | 3,385,378 | 4,575,311 | 4,234,459 | 5,333,467 |
| Guangdong Province | | Foshan City | 2,769,289 | 1,367,481 | 1,447,617 | 1,503,435 | 2,100,283 |
| Guangdong Province | | Guangzhou City | 7,431,012 | 7,883,336 | 8,496,046 | 8,040,598 | 8,814,930 |
| Guangdong Province | | Heyuan City | 546,248 | 622,778 | 707,243 | 776,728 | 916,946 |
| Guangdong Province | | Huizhou | 1,739,446 | 2,022,727 | 2,425,318 | 2,673,762 | 3,250,534 |
| Guangdong Province | | Jiangmen | 1,961,260 | 1,706,368 | 1,634,335 | 1,441,107 | 2,023,158 |
| Guangdong Province | | Jieyang | 2,685,382 | 2,668,461 | 2,891,398 | 2,521,638 | 2,934,889 |
| Guangdong Province | | Maoming | 1,787,654 | 1,878,424 | 1,977,709 | 2,062,203 | 2,191,600 |
| Guangdong Province | | Meizhou | 1,125,470 | 1,199,787 | 1,193,684 | 1,262,729 | 1,359,094 |
| Guangdong Province | | Qingyuan | 664,735 | 635,539 | 702,674 | 695,233 | 792,695 |
| Guangdong Province | | Shan Tou | 3,388,313 | 3,370,834 | 3,377,157 | 3,593,630 | 3,855,108 |
| Guangdong Province | | Shanwei City | 1,296,838 | 1,437,869 | 1,555,740 | 1,732,291 | 1,886,605 |
| Guangdong Province | | Shaoguan City | 1,146,512 | 1,145,114 | 1,082,968 | 1,108,838 | 1,090,256 |
| Guangdong Province | | Shenzhen | 6,044,828 | 6,355,337 | 8,890,277 | 10,374,797 | 14,008,131 |
| Guangdong Province | | Yangjiang | 670,852 | 708,502 | 745,357 | 771,425 | 828,866 |
| Guangdong Province | | Yunfu City | 616,289 | 631,753 | 659,365 | 679,133 | 718,706 |
| Guangdong Province | | Zhangjiang City | 1,964,820 | 2,091,223 | 2,255,917 | 2,374,737 | 2,572,765 |
| Guangdong Province | | Zhaoqing | 1,121,679 | 921,561 | 1,063,965 | 933,614 | 1,118,936 |
| Guangdong Province | | Zhongshan City | 1,628,596 | 1,388,404 | 1,376,039 | 1,169,044 | 1,523,274 |
| Guangdong Province | | Zhuhai City | 728,281 | 773,844 | 873,206 | 935,931 | 1,137,018 |
| Guangxi Zhuang Autonomous Region | | Baise | 290,189 | 293,385 | 298,454 | 298,953 | 303,543 |
| Guangxi Zhuang Autonomous Region | | Beihai | 684,226 | 656,640 | 650,990 | 697,951 | 754,385 |
| Guangxi Zhuang Autonomous Region | | Chongzuo | 251,456 | 250,838 | 250,222 | 248,569 | 247,031 |
| Guangxi Zhuang Autonomous Region | | Fangchenggang | 125,714 | 133,465 | 141,639 | 150,080 | 170,074 |
| Guangxi Zhuang Autonomous Region | | Guigang | 561,864 | 580,102 | 601,334 | 577,499 | 637,289 |
| Guangxi Zhuang Autonomous Region | | Guilin | 1,299,603 | 1,200,154 | 1,158,133 | 1,066,924 | 1,292,929 |
| Guangxi Zhuang Autonomous Region | | Hechi City | 303,256 | 277,921 | 277,348 | 255,632 | 268,221 |
| Guangxi Zhuang Autonomous Region | | Hezhou | 303,219 | 245,264 | 283,582 | 250,476 | 238,318 |
| Guangxi Zhuang Autonomous Region | | Laibin City | 344,794 | 344,425 | 346,258 | 307,322 | 337,658 |
| Guangxi Zhuang Autonomous Region | | Liuzhou | 1,561,348 | 1,229,068 | 1,399,015 | 959,457 | 1,393,927 |
| Guangxi Zhuang Autonomous Region | | Nanning City | 2,839,437 | 2,759,034 | 2,305,285 | 1,644,899 | 2,278,692 |
| Guangxi Zhuang Autonomous Region | | Qinzhou | 290,925 | 299,401 | 309,346 | 258,813 | 310,722 |
| Guangxi Zhuang Autonomous Region | | Wuzhou | 601,334 | 608,850 | 617,629 | 583,857 | 592,070 |
| Guangxi Zhuang Autonomous Region | | Yulin City | 1,155,581 | 1,127,335 | 1,185,871 | 1,020,175 | 1,127,436 |
| Hainan Province | Baisha Li Autonomous County | | 11 | 11 | 11 | 12 | 12 |
| Hainan Province | Baoting Li and Miao Autonomous County | | 4,981 | 5,144 | 5,313 | 5,487 | 5,667 |
| Hainan Province | Changjiang Li Autonomous County | | 60,402 | 62,384 | 64,431 | 66,546 | 68,730 |
| Hainan Province | Chengmai County | | 70,097 | 73,007 | 75,969 | 79,534 | 82,212 |
| Hainan Province | Danzhou | | 256,560 | 265,616 | 272,572 | 279,841 | 294,128 |
| Hainan Province | Ding'an County | | 51,505 | 53,246 | 55,053 | 56,929 | 58,878 |
| Hainan Province | Dongfang City | | 120,700 | 123,260 | 125,891 | 132,293 | 135,548 |
| Hainan Province | Haikou | | 1,299,706 | 1,510,075 | 1,636,846 | 1,950,283 | 2,328,295 |
| Hainan Province | Ledong Li Autonomous County | | 78,622 | 81,202 | 83,867 | 86,620 | 89,460 |
| Hainan Province | Lingao County | | 66,533 | 68,639 | 70,961 | 74,023 | 75,305 |
| Hainan Province | Lingshui Li Autonomous County | | 75,787 | 77,738 | 80,844 | 77,097 | 69,956 |
| Hainan Province | Qionghai City | | 143,749 | 148,466 | 153,339 | 158,372 | 163,569 |
| Hainan Province | Qiongzhong Li and Miao Autonomous County | | 4,698 | 4,852 | 5,012 | 5,176 | 5,346 |
| Hainan Province | Sansha | |  |  |  |  |  |
| Hainan Province | Sanya | | 195,067 | 222,216 | 255,330 | 297,494 | 346,660 |
| Hainan Province | Tunchang County | | 36,647 | 37,850 | 39,092 | 40,375 | 41,700 |
| Hainan Province | Wanning | | 116,019 | 120,158 | 123,806 | 128,094 | 131,288 |
| Hainan Province | Wenchang City | | 29,577 | 30,579 | 31,618 | 32,698 | 33,819 |
| Hainan Province | Wuzhishan City | | 22,691 | 23,436 | 24,205 | 25,000 | 25,820 |
| Hong Kong Special Administrative Region | Hong Kong Special Administrative Region | | 3,740,298 |  | 4,418,702 |  |  |
| Macao Special Administrative Region | Macao Special Administrative Region | | 153,853 |  | 203,606 |  |  |
| Chongqing | Chongqing | | 9,190,119 | 9,006,175 | 8,277,629 | 7,263,348 | 8,189,438 |
| Guizhou Province | Anshun City | | 492,564 | 488,989 | 485,440 | 458,242 | 477,268 |
| Guizhou Province | Bijie City | | 924,203 | 938,396 | 955,112 | 966,953 | 985,078 |
| Guizhou Province | Guiyang City | | 2,496,593 | 2,540,533 | 2,902,640 | 2,365,131 | 3,456,073 |
| Guizhou Province | Liupanshui | | 854,307 | 870,777 | 887,516 | 904,682 | 922,131 |
| Guizhou Province | Qiandongnan Miao and Dong Autonomous Prefecture | | 511,820 | 486,808 | 463,376 | 407,087 | 409,622 |
| Guizhou Province | Qiannan Buyi and Miao Autonomous Prefecture | | 501,816 | 482,268 | 469,802 | 396,579 | 424,142 |
| Guizhou Province | Qianxinan Buyi and Miao Autonomous Prefecture | | 456,908 | 455,177 | 450,388 | 436,291 | 439,307 |
| Guizhou Province | Tongren City | | 355,360 | 343,925 | 329,362 | 309,567 | 306,316 |
| Guizhou Province | Zunyi City | | 1,308,829 | 1,184,152 | 1,143,976 | 1,153,581 | 1,129,099 |
| Sichuan Province | Aba Tibetan and Qiang Autonomous Prefecture | | 10,778 | 11,099 | 11,280 | 11,770 | 11,671 |
| Sichuan Province | Bazhong City | | 398,721 | 398,351 | 375,709 | 397,613 | 397,244 |
| Sichuan Province | Chengdu | | 4,980,531 | 6,308,536 | 4,361,641 | 6,766,077 | 10,363,501 |
| Sichuan Province | Dazhou | | 1,389,347 | 1,413,268 | 1,226,805 | 1,239,124 | 1,132,072 |
| Sichuan Province | Deyang City | | 1,298,386 | 995,672 | 726,136 | 970,783 | 963,507 |
| Sichuan Province | Ganzi Tibetan Autonomous Prefecture | | 3,222 | 3,351 | 3,797 | 4,198 | 4,676 |
| Sichuan Province | Guang'an | | 1,029,071 | 909,881 | 730,390 | 592,437 | 557,784 |
| Sichuan Province | Guangyuan City | | 581,887 | 524,020 | 469,673 | 423,901 | 382,407 |
| Sichuan Province | Leshan | | 899,787 | 864,705 | 758,612 | 876,794 | 915,427 |
| Sichuan Province | Liangshan Yi Autonomous Prefecture | | 569,620 | 600,290 | 632,611 | 655,872 | 682,478 |
| Sichuan Province | Luzhou | | 826,161 | 836,830 | 842,611 | 689,307 | 736,928 |
| Sichuan Province | Meishan City | | 444,737 | 582,127 | 218,695 | 280,382 | 275,281 |
| Sichuan Province | Mianyang City | | 1,739,775 | 1,605,341 | 1,073,844 | 1,325,666 | 1,435,287 |
| Sichuan Province | Nanchong | | 1,680,145 | 1,685,509 | 1,426,798 | 1,547,918 | 1,484,410 |
| Sichuan Province | Neijiang City | | 1,411,461 | 1,328,391 | 1,009,627 | 1,153,696 | 1,080,834 |
| Sichuan Province | Panzhihua | | 718,008 | 757,210 | 798,550 | 842,147 | 888,125 |
| Sichuan Province | Suining City | | 916,569 | 1,067,821 | 882,627 | 877,538 | 866,288 |
| Sichuan Province | Ya'an City | | 347,656 | 336,114 | 341,782 | 336,910 | 339,390 |
| Sichuan Province | Yibin | | 1,006,574 | 950,009 | 905,076 | 883,522 | 845,297 |
| Sichuan Province | Zigong City | | 891,441 | 923,533 | 745,549 | 841,404 | 800,777 |
| Sichuan Province | Ziyang City | | 762,018 | 674,159 | 565,215 | 502,774 | 459,092 |
| Tibet Autonomous Region | Lhasa | | 198,867 | 215,924 | 234,444 | 252,126 | 269,449 |
| Tibet Autonomous Region | Nagqu | | 1,296 | 1,455 | 1,634 | 1,835 | 1,952 |
| Tibet Autonomous Region | Ngari Area | | 82 | 91 | 101 | 112 | 125 |
| Tibet Autonomous Region | Nyingchi | | 3,449 | 3,743 | 4,223 | 4,547 | 4,943 |
| Tibet Autonomous Region | Qamdo | | 9,125 | 9,656 | 10,251 | 10,853 | 11,492 |
| Tibet Autonomous Region | Shannan City | | 18,357 | 18,668 | 18,985 | 19,232 | 19,633 |
| Tibet Autonomous Region | Shigatse | | 19,075 | 20,075 | 21,127 | 22,235 | 23,204 |
| Yunnan Province | Baoshan | | 248,479 | 256,712 | 260,366 | 257,708 | 275,109 |
| Yunnan Province | Chuxiong Yi Autonomous Prefecture | | 310,599 | 319,152 | 327,942 | 333,711 | 340,463 |
| Yunnan Province | Dali Bai Autonomous Prefecture | | 420,245 | 430,313 | 440,620 | 451,174 | 459,088 |
| Yunnan Province | Dehong Dai and Jingpo Autonomous Prefecture | | 155,145 | 164,570 | 164,911 | 163,857 | 180,878 |
| Yunnan Province | Diqing Tibetan Autonomous Prefecture | | 2,929 | 3,114 | 3,311 | 3,521 | 3,461 |
| Yunnan Province | Honghe Hani and Yi Autonomous Prefecture | | 801,514 | 836,419 | 872,135 | 908,063 | 945,856 |
| Yunnan Province | Kunming | | 3,743,273 | 3,948,569 | 4,115,030 | 4,269,421 | 4,542,777 |
| Yunnan Province | Lijiang | | 194,283 | 204,185 | 214,592 | 225,530 | 235,427 |
| Yunnan Province | Lincang | | 169,919 | 173,414 | 176,980 | 177,960 | 177,765 |
| Yunnan Province | Nujiang Lisu Autonomous Prefecture | | 18,829 | 19,626 | 20,456 | 21,322 | 22,225 |
| Yunnan Province | Pu'er City | | 146,032 | 147,873 | 149,737 | 151,394 | 152,910 |
| Yunnan Province | Qujing City | | 1,114,689 | 1,153,623 | 1,193,925 | 1,214,740 | 1,235,003 |
| Yunnan Province | Wenshan Zhuang and Miao Autonomous Prefecture | | 224,687 | 233,103 | 241,834 | 249,239 | 257,025 |
| Yunnan Province | Xishuangbanna Dai Autonomous Prefecture | | 74,064 | 79,115 | 84,510 | 89,716 | 96,430 |
| Yunnan Province | Yuxi City | | 645,193 | 680,103 | 716,903 | 747,705 | 792,992 |
| Yunnan Province | Zhaotong | | 523,354 | 557,479 | 593,448 | 632,595 | 673,887 |
| Gansu Province | Baiyin City | | 549,329 | 547,705 | 546,094 | 544,276 | 541,746 |
| Gansu Province | Dingxi | | 423,064 | 413,892 | 404,922 | 395,996 | 386,500 |
| Gansu Province | Gannan Tibetan Autonomous Prefecture | | 46,879 | 48,642 | 50,470 | 52,367 | 54,335 |
| Gansu Province | Jiayuguan | | 153,429 | 164,763 | 158,429 | 156,739 | 165,774 |
| Gansu Province | Jinchang City | | 140,114 | 142,037 | 143,987 | 145,962 | 146,860 |
| Gansu Province | Jiuquan City | | 284,050 | 300,721 | 317,214 | 336,659 | 354,957 |
| Gansu Province | Lan'Zhou City | | 2,621,843 | 2,793,833 | 2,955,763 | 3,150,133 | 3,280,223 |
| Gansu Province | Linxia Hui Autonomous Prefecture | | 434,047 | 448,096 | 462,612 | 477,609 | 492,673 |
| Gansu Province | Longnan City | | 236,707 | 235,884 | 235,066 | 234,251 | 233,439 |
| Gansu Province | Pingliang City | | 553,449 | 553,521 | 553,596 | 551,872 | 551,670 |
| Gansu Province | Qingyang City | | 520,038 | 497,063 | 475,107 | 454,124 | 410,286 |
| Gansu Province | Tianshui City | | 1,029,512 | 1,036,396 | 1,042,475 | 1,043,951 | 1,042,227 |
| Gansu Province | Wuwei City | | 376,837 | 374,582 | 372,340 | 369,711 | 367,583 |
| Gansu Province | Zhangye City | | 362,335 | 354,695 | 347,214 | 338,328 | 330,310 |
| Ningxia Hui Autonomous Region | Guyuan City | | 175,348 | 165,217 | 155,671 | 144,837 | 135,041 |
| Ningxia Hui Autonomous Region | Shizuishan City | | 476,731 | 495,183 | 513,474 | 531,390 | 551,512 |
| Ningxia Hui Autonomous Region | Wuzhong City | | 470,347 | 510,206 | 550,562 | 599,911 | 603,041 |
| Ningxia Hui Autonomous Region | Yinchuan | | 1,280,216 | 1,512,473 | 1,780,164 | 2,068,188 | 2,387,270 |
| Ningxia Hui Autonomous Region | Zhongwei City | | 248,589 | 270,367 | 294,056 | 319,745 | 343,078 |
| Qinghai Province | Golog Tibetan Autonomous Prefecture | | 82 | 94 | 107 | 123 | 141 |
| Qinghai Province | Haibei Tibetan Autonomous Prefecture | | 5,905 | 6,066 | 6,232 | 6,402 | 6,577 |
| Qinghai Province | Haidong City | | 319,996 | 322,672 | 325,509 | 328,571 | 327,152 |
| Qinghai Province | Hainan Tibetan Autonomous Prefecture | | 11,758 | 12,747 | 13,832 | 15,005 | 16,271 |
| Qinghai Province | Haixi Mongolian and Tibetan Autonomous Prefecture | | 77,755 | 94,724 | 114,988 | 139,559 | 169,289 |
| Qinghai Province | Huangnan Tibetan Autonomous Prefecture | | 26,879 | 28,042 | 29,266 | 30,606 | 32,067 |
| Qinghai Province | Xining | | 1,335,152 | 1,458,758 | 1,593,637 | 1,739,664 | 1,863,158 |
| Qinghai Province | Yushu Tibetan Autonomous Prefecture | | 285 | 342 | 411 | 493 | 592 |
| Shaanxi Province | Ankang | | 605,496 | 607,339 | 595,026 | 596,732 | 612,903 |
| Shaanxi Province | Baoji | | 1,603,738 | 1,630,992 | 1,657,655 | 1,682,485 | 1,714,149 |
| Shaanxi Province | Hanzhong | | 1,242,256 | 1,231,407 | 1,060,245 | 991,415 | 1,160,514 |
| Shaanxi Province | Shangluo | | 281,098 | 280,920 | 280,743 | 280,568 | 280,395 |
| Shaanxi Province | Tongchuan City | | 393,676 | 403,791 | 414,169 | 424,817 | 435,239 |
| Shaanxi Province | Weinan City | | 2,209,142 | 2,199,760 | 2,207,842 | 2,200,549 | 2,165,012 |
| Shaanxi Province | Xi'an | | 6,002,161 | 4,126,939 | 4,078,800 | 5,338,636 | 7,675,322 |
| Shaanxi Province | Xianyang | | 2,237,005 | 2,286,136 | 2,208,614 | 2,184,072 | 2,264,993 |
| Shaanxi Province | Yan'an | | 467,182 | 486,498 | 506,612 | 527,559 | 549,371 |
| Shaanxi Province | Yulin | | 779,166 | 779,928 | 780,022 | 772,273 | 760,404 |
| Xinjiang Uygur Autonomous Region | Aksu Area | | 632,284 | 665,146 | 700,664 | 730,937 | 772,433 |
| Xinjiang Uygur Autonomous Region | Altay Region | | 56,016 | 58,054 | 60,166 | 62,355 | 64,624 |
| Xinjiang Uygur Autonomous Region | Aral | | 50,951 | 58,039 | 66,132 | 75,267 | 85,918 |
| Xinjiang Uygur Autonomous Region | Bayingolin Mongolian Autonomous Prefecture | | 512,570 | 586,116 | 633,837 | 658,524 | 787,045 |
| Xinjiang Uygur Autonomous Region | Beitun City | | 16,218 | 16,808 | 17,419 | 18,053 | 18,710 |
| Xinjiang Uygur Autonomous Region | Bortala Mongolian Autonomous Prefecture | | 69,294 | 70,966 | 72,523 | 74,121 | 75,654 |
| Xinjiang Uygur Autonomous Region | Changji Hui Autonomous Prefecture | | 712,647 | 745,432 | 780,309 | 813,132 | 826,837 |
| Xinjiang Uygur Autonomous Region | Hami City | | 274,587 | 296,145 | 319,396 | 341,173 | 367,583 |
| Xinjiang Uygur Autonomous Region | Hotan Area | | 319,563 | 349,804 | 382,886 | 418,093 | 458,733 |
| Xinjiang Uygur Autonomous Region | Ili Kazakh Autonomous Prefecture | | 850,202 | 871,003 | 891,839 | 893,188 | 920,431 |
| Xinjiang Uygur Autonomous Region | Karamay | | 263,383 | 314,865 | 368,938 | 430,365 | 503,732 |
| Xinjiang Uygur Autonomous Region | Kashgar Area | | 944,399 | 1,020,836 | 1,103,460 | 1,192,771 | 1,289,310 |
| Xinjiang Uygur Autonomous Region | Kizilsu Kirgiz Autonomous Prefecture | | 66,710 | 72,935 | 79,741 | 87,181 | 95,316 |
| Xinjiang Uygur Autonomous Region | Kokkhara | | 17,743 | 18,167 | 18,602 | 19,047 | 19,503 |
| Xinjiang Uygur Autonomous Region | Kunyu City | | 468 | 512 | 560 | 613 | 671 |
| Xinjiang Uygur Autonomous Region | Shihezi City | | 288,736 | 329,874 | 375,897 | 401,849 | 474,131 |
| Xinjiang Uygur Autonomous Region | Shuanghe City | | 13,697 | 14,011 | 14,332 | 14,660 | 14,996 |
| Xinjiang Uygur Autonomous Region | Tacheng Area | | 237,665 | 275,864 | 325,588 | 373,218 | 438,158 |
| Xinjiang Uygur Autonomous Region | The Seventh Division Of The Production and Construction Corps | | 2,360 | 2,765 | 3,239 | 3,796 | 4,448 |
| Xinjiang Uygur Autonomous Region | Tiemenguan City | | 9,760 | 10,734 | 11,805 | 12,984 | 14,279 |
| Xinjiang Uygur Autonomous Region | Tumusuk | | 960 | 1,089 | 1,236 | 1,404 | 1,595 |
| Xinjiang Uygur Autonomous Region | Turpan | | 133,447 | 142,003 | 151,019 | 160,161 | 170,926 |
| Xinjiang Uygur Autonomous Region | Urumqi | | 2,883,594 | 3,376,096 | 3,927,175 | 4,260,879 | 5,302,147 |
| Xinjiang Uygur Autonomous Region | Wujiaqu | | 54,941 | 62,776 | 71,734 | 81,974 | 93,680 |

**Table S2. Relative risk summary for the association between NDVI and mortality.**

| **Author** | **Country** | **Relative risk (95% CI)** | **Exposure unit reported, NDVI score** | **Health outcome** | **Age group** | **Syudy design** |
| --- | --- | --- | --- | --- | --- | --- |
| Ji et al. (2019) | China | 0·950 (0·945–0·955) | per 0.1 unit increase | All-cause mortality | > = 80 years | Cohort study |
| Ji et al. (2020) | China | 0.926 (0.885–0.971) | per 0.1 unit increase | All-cause mortality | > = 65 years | Cohort study |
| Zhang et al. (2021) | China | 0.926 (0.917–0.935) | per 0.1 unit increase | All-cause mortality | > = 65 years | Cohort study |
| Li et al. (2023) | China | 0.976 (0.957–0.996) | per 0.1 unit increase | All-cause mortality | / | Ecological study |
| Rojas et al. (2019) | / | 0.96 (0.95–0.97) | per 0.1 unit increase | All-cause mortality | > = 20 years | Meta-analysis |
| Yuan et al. (2020) | / | 0.99 (0.97, 1.00) | per 0.1 unit increase | All-cause mortality | > = 60 years | Meta-analysis |

**Table S3. Point Estimates and Confidence Intervals from the uncertainty analysis for different scenarios**

| **Scenarios** | **Region** | **Mean** | **Median** | **Pct2.5** | **Pct97.5** |
| --- | --- | --- | --- | --- | --- |
| NDVI Change (2000-2010) | East China | 64 | 39 | -125 | 347 |
|  | North China | 34 | 10 | -314 | 454 |
|  | Northeast China | -11 | -3 | -349 | 294 |
|  | Northwest China | -10 | -2 | -186 | 137 |
|  | South China | -24 | -8 | -231 | 119 |
|  | Southwest China | -5 | -1 | -294 | 256 |
|  | Central China | 18 | 7 | -283 | 348 |
| NDVI Change (2010-2020) | East China | -86 | -47 | -483 | 164 |
|  | North China | -229 | -147 | -1,364 | 597 |
|  | Northeast China | 25 | 8 | -339 | 439 |
|  | Northwest China | -36 | -14 | -294 | 138 |
|  | South China | -14 | -3 | -345 | 286 |
|  | Southwest China | -42 | -28 | -265 | 120 |
|  | Central China | -154 | -110 | -653 | 139 |
| NDVI Difference (Counterfactual scenarios, 2000) | East China | -115 | -47 | -912 | 448 |
|  | North China | -290 | -112 | -2,529 | 1,128 |
|  | Northeast China | 30 | 12 | -780 | 882 |
|  | Northwest China | -47 | -14 | -571 | 336 |
|  | South China | -18 | -2 | -609 | 495 |
|  | Southwest China | -54 | -12 | -776 | 509 |
|  | Central China | -193 | -127 | -893 | 209 |
| NDVI Difference (Counterfactual scenarios, 2010) | East China | -97 | -34 | -874 | 461 |
|  | North China | -219 | -99 | -1,648 | 791 |
|  | Northeast China | 21 | 6 | -745 | 786 |
|  | Northwest China | -37 | -9 | -450 | 278 |
|  | South China | -14 | -1 | -499 | 417 |
|  | Southwest China | -44 | -12 | -685 | 455 |
|  | Central China | -149 | -90 | -789 | 233 |

**Table S4. Results of the sensitivity analysis employing distinct NDVI.**

| **Analysis (NDVI Source)** | **Scenarios** | **Deaths/Averted deaths in China** | **95%CI lower** | **95%CI upper** | **Change** |
| --- | --- | --- | --- | --- | --- |
| Main analysis (using the annual median population-weighted NDVI) | NDVI Change (2000-2010) | 9,951 | 3,346 | 18,106 | / |
|  | NDVI Change (2010-2020) | -37,653 | -60,135 | -26,327 | / |
|  | NDVI Difference  (Counterfactual scenarios, 2000) | -110,976 | -82,010 | -171,561 | / |
|  | NDVI Difference  (Counterfactual scenarios, 2010) | -118,330 | -87,362 | -183,283 | / |
| Sensitivity analysis (using the annual median unweighted NDVI) | NDVI Change (2000-2010) | 7,281 | 5,482 | 10,834 | -27% |
|  | NDVI Change (2010-2020) | -35,176 | -26,116 | -53,861 | -7% |
|  | NDVI Difference  (Counterfactual scenarios, 2000) | -112,231 | -82,860 | -173,842 | 1% |
|  | NDVI Difference  (Counterfactual scenarios, 2010) | -128,436 | -94,684 | -199,546 | 9% |
| Sensitivity analysis (using the mean NDVI from June to August) | NDVI Change (2000-2010) | -24,499 | -18,179 | -37,554 | -346% |
|  | NDVI Change (2010-2020) | -32,934 | -24,479 | -50,312 | -13% |
|  | NDVI Difference  (Counterfactual scenarios, 2000) | -81,688 | -60,286 | -126,651 | -26% |
|  | NDVI Difference  (Counterfactual scenarios, 2010) | -75,098 | -55,411 | -116,484 | -37% |

**Table S5. Results of the sensitivity analysis employing distinct ERFs.**

| **Reference** | **Relative risk (95% CI)** | **Deaths/Averted deaths in China** | **95%CI lower** | **95%CI upper** | **Change** |
| --- | --- | --- | --- | --- | --- |
| **Rojas et al. (2019)**  **(Main analysis)** | 0.96 (0.94–0.97) | 9,951 | 3,346 | 18,106 | / |
| Ji et al. (2020) | 0.926 (0.885–0.971) | 17,542 | 6,922 | 27,061 | 76% |
| Zhang et al. (2021) | 0.926 (0.917–0.935) | 17,542 | 15,431 | 19,646 | 76% |
| Li et al. (2023) | 0.976 (0.957–0.996) | 5,732 | 958 | 10,243 | -42% |
| Ji et al. (2019) | 0·950 (0·945–0·955) | 11,898 | 10,716 | 13,077 | 20% |
| Yuan et al. (2020) | 0.99 (0.97, 1.00) | 2,393 | 0 | 7,159 | -76% |
| **Rojas et al. (2019)**  **(Main analysis)** | 0.96 (0.94–0.97) | -37,653 | -60,135 | -26,327 | / |
| Ji et al. (2020) | 0.926 (0.885–0.971) | -58,168 | -21,794 | -94,356 | 54% |
| Zhang et al. (2021) | 0.926 (0.917–0.935) | -58,168 | -50,628 | -65,849 | 54% |
| Li et al. (2023) | 0.976 (0.957–0.996) | -17,949 | -2,934 | -32,762 | -52% |
| Ji et al. (2019) | 0·950 (0·945–0·955) | -38,362 | -34,354 | -42,409 | 2% |
| Yuan et al. (2020) | 0.99 (0.97, 1.00) | -7,378 | 0 | -22,567 | -80% |
| **Rojas et al. (2019)**  **(Main analysis)** | 0.96 (0.94–0.97) | -110,976 | -82,010 | -171,561 | / |
| Ji et al. (2020) | 0.926 (0.885–0.971) | -216,213 | -79,160 | -171,741 | 95% |
| Zhang et al. (2021) | 0.926 (0.917–0.935) | -216,213 | -187,289 | -245,954 | 95% |
| Li et al. (2023) | 0.976 (0.957–0.996) | -65,035 | -10,532 | -119,834 | -41% |
| Ji et al. (2019) | 0·950 (0·945–0·955) | -140,814 | -125,783 | -156,071 | 27% |
| Yuan et al. (2020) | 0.99 (0.97, 1.00) | -26,555 | 0 | -82,010 | -76% |
| **Rojas et al. (2019)**  **(Main analysis)** | 0.96 (0.94–0.97) | -118,330 | -87,362 | -183,283 | / |
| Ji et al. (2020) | 0.926 (0.885–0.971) | -231,305 | -84,318 | -385,500 | 95% |
| Zhang et al. (2021) | 0.926 (0.917–0.935) | -231,305 | -200,183 | -263,360 | 95% |
| Li et al. (2023) | 0.976 (0.957–0.996) | -69,240 | -11,192 | -127,811 | -41% |
| Ji et al. (2019) | 0·950 (0·945–0·955) | -150,289 | -134,183 | -166,654 | 27% |
| Yuan et al. (2020) | 0.99 (0.97, 1.00) | -28,236 | 0 | -87,362 | -76% |

**References**

1. Horizon7. Description of administrative district data [Internet]. [cited 2022 Jun 29]. Available from: <http://horizon2021.xyz/archives/1057>
2. Balk, D. L., Deichmann, U., Yetman, G., Pozzi, F., Hay, S. I., & Nelson, A. (2006). Determining global population distribution: methods, applications and data. Advances in parasitology, 62, 119-156.
3. Center for International Earth Science Information Network - CIESIN - Columbia University, International Food Policy Research Institute - IFPRI, The World Bank, Centro Internacional de Agricultura Tropical - CIAT. Global Rural-Urban Mapping Project, Version 1 (GRUMPv1): Urban Extent Polygons, Revision 02 [Internet]. Palisades, New York: NASA Socioeconomic Data and Applications Center (SEDAC); 2021. Available from: <https://doi.org/10.7927/np6p-qe61>
4. Center for International Earth Science Information Network - CIESIN - Columbia University. Gridded Population of the World, Version 4 (GPWv4): Population Density, Revision 11 [Internet]. Palisades, New York: NASA Socioeconomic Data and Applications Center (SEDAC); 2018. Available from: <https://doi.org/10.7927/H49C6VHW>
5. Mueller, N., Nieuwenhuijsen, M. J., & Rojas-Rueda, D. (2020). Quantitative health impact and burden of disease assessment of traffic-related air pollution. In Traffic-Related Air Pollution (pp. 339-359). Elsevier.
6. Mueller, N., Rojas-Rueda, D., Basagaña, X., Cirach, M., Cole-Hunter, T., Dadvand, P., ... & Nieuwenhuijsen, M. (2017). Health impacts related to urban and transport planning: A burden of disease assessment. Environment international, 107, 243-257.
7. Rojas-Rueda, D., Nieuwenhuijsen, M. J., Gascon, M., Perez-Leon, D., & Mudu, P. (2019). Green spaces and mortality: a systematic review and meta-analysis of cohort studies. The Lancet Planetary Health, 3(11), e469-e477.
